# The Human Urine Metabolome

**DOI:** 10.1371/journal.pone.0073076

**Published:** 2013-09-04

**Authors:** Souhaila Bouatra, Farid Aziat, Rupasri Mandal, An Chi Guo, Michael R. Wilson, Craig Knox, Trent C. Bjorndahl, Ramanarayan Krishnamurthy, Fozia Saleem, Philip Liu, Zerihun T. Dame, Jenna Poelzer, Jessica Huynh, Faizath S. Yallou, Nick Psychogios, Edison Dong, Ralf Bogumil, Cornelia Roehring, David S. Wishart

**Affiliations:** 1 Department of Biological Sciences, University of Alberta, Edmonton, Alberta, Canada; 2 Department of Computing Sciences, University of Alberta, Edmonton, Alberta, Canada; 3 Cardiovascular Research Center, Massachusetts General Hospital, Harvard Medical School, Boston, Massachusetts, United States of America; 4 BIOCRATES Life Sciences AG, Innsbruck, Austria; 5 National Institute for Nanotechnology, Edmonton, Alberta, Canada; Mayo Clinic, United States of America

## Abstract

Urine has long been a “favored” biofluid among metabolomics researchers. It is sterile, easy-to-obtain in large volumes, largely free from interfering proteins or lipids and chemically complex. However, this chemical complexity has also made urine a particularly difficult substrate to fully understand. As a biological waste material, urine typically contains metabolic breakdown products from a wide range of foods, drinks, drugs, environmental contaminants, endogenous waste metabolites and bacterial by-products. Many of these compounds are poorly characterized and poorly understood. In an effort to improve our understanding of this biofluid we have undertaken a comprehensive, quantitative, metabolome-wide characterization of human urine. This involved both computer-aided literature mining and comprehensive, quantitative experimental assessment/validation. The experimental portion employed NMR spectroscopy, gas chromatography mass spectrometry (GC-MS), direct flow injection mass spectrometry (DFI/LC-MS/MS), inductively coupled plasma mass spectrometry (ICP-MS) and high performance liquid chromatography (HPLC) experiments performed on multiple human urine samples. This multi-platform metabolomic analysis allowed us to identify 445 and quantify 378 unique urine metabolites or metabolite species. The different analytical platforms were able to identify (quantify) a total of: 209 (209) by NMR, 179 (85) by GC-MS, 127 (127) by DFI/LC-MS/MS, 40 (40) by ICP-MS and 10 (10) by HPLC. Our use of multiple metabolomics platforms and technologies allowed us to identify several previously unknown urine metabolites and to substantially enhance the level of metabolome coverage. It also allowed us to critically assess the relative strengths and weaknesses of different platforms or technologies. The literature review led to the identification and annotation of another 2206 urinary compounds and was used to help guide the subsequent experimental studies. An online database containing the complete set of 2651 confirmed human urine metabolite species, their structures (3079 in total), concentrations, related literature references and links to their known disease associations are freely available at http://www.urinemetabolome.ca.

## Introduction

Metabolomics is a relatively young branch of “omics” science concerned with the systematic study of the chemical products or metabolites that cells and organisms generate. Because metabolites are the downstream products of numerous genome-wide or proteome-wide interactions, the metabolome (the sum of all metabolites in an organism) can be a very sensitive measure of an organism’s phenotype. This fact has made metabolomics particularly useful in the study of environment-gene interactions [1], [2], [3], [4], the identification of disease biomarkers [5], [6], [7], [8], [9] and the discovery of drugs [10]. Unlike its older “omics” cousins, where complete or near-complete coverage of the genome or proteome is fairly routine, metabolomics still struggles to cover even a tiny fraction of the metabolome. Indeed, most human metabolomic studies published today, even those exploiting the latest and most sensitive LC-MS/MS technologies, typically succeed in identifying or characterizing fewer than 100 compounds [11], [12], [13], [14]. This corresponds to less than 1% of the known human metabolome [15], [16]. In an effort to help improve this situation, we (and others) have started to undertake the systematic characterization of various human biofluid metabolomes. This includes the human cerebrospinal fluid metabolome [17], [18], the human saliva metabolome [19], and the human serum metabolome [20]. We have now turned our attention to characterizing the human urine metabolome.

Urine, as produced by mammals, is a transparent, sterile, amber-colored fluid generated by the kidneys. The kidneys extract the soluble wastes from the bloodstream, as well as excess water, sugars, and a variety of other compounds. The resulting urine contains high concentrations of urea (from amino acid metabolism), inorganic salts (chloride, sodium, and potassium), creatinine, ammonia, organic acids, various water-soluble toxins and pigmented products of hemoglobin breakdown, including urobilin, which gives urine its characteristic color. Urination is the primary route by which the body eliminates water-soluble waste products. The average adult generates between 1.5–2.0 liters of urine per day, which over the course of their lifetime would be enough to fill a small backyard swimming pool (5 X 8 X 1.5 m).

While largely viewed as a waste product, urine has considerable value as a diagnostic biofluid. Indeed the analysis of urine for medical purposes dates back to ancient Egypt [21], [22], [23], [24]. Hippocrates largely legitimized the medical practice of uroscopy (the study of urine for medical diagnostics) where examination of the color, cloudiness, smell and even the taste of urine was used to identify a variety of diseases. Throughout the Byzantine era and well into the Middle Ages, urine color wheels (a diagram that linked the color of urine to a particular disease) were commonly used by physicians to make diagnoses [21], [25]. A brownish color would indicate jaundice, a red hue (blood) might indicate urinary tract tumors, absence of color would be indicative of diabetes and foamy urine would indicate proteinuria. With the advent of modern clinical techniques in the middle of the 19^th^ century, uroscopy largely disappeared. However, urine has continued to be an important cornerstone to modern medical practice. In fact, it was the first biofluid to be used to clinically diagnose a human genetic disease - alkaptonuria [26]. Even today urine analysis is routinely performed with dipstick tests that can readily measure urinary glucose, bilirubin, ketone bodies, nitrates, leukocyte esterase, specific gravity, hemoglobin, urobilinogen and protein. More detailed urinalysis can be also used to study a variety of renal conditions, such as bladder, ovarian and kidney diseases [27], [28], [29], [30].

Being an important and easily accessible biological fluid, urine has been the subject of detailed chemical analysis for more than 100 years [31], [32]. Extensive tables of normal reference ranges have been published for more than 100 urine ions and metabolites [33], [34], [35], [36], [37]. In addition to these referential clinical chemistry studies, several groups have applied various “global” metabolomic or metabolite profiling methods to urine, such as high resolution nuclear magnetic resolution (NMR) spectroscopy [38], [39], [40], high performance liquid chromatography (HPLC) [41], high performance liquid chromatography-tandem mass spectrometry (HPLC-MS/MS) [42], ultra high performance liquid chromatography-high resolution orbitrap mass spectrometry (UHPLC-HRMS) [43], ultra high performance liquid chromatography (UPLC-RP) [31], [44], fast atom bombardment ionization coupled with mass spectroscopy (FAB-MS) [35], two-dimensional gas chromatography coupled to quadrupole mass spectrometry (GCxGC-MS) [32] or time-of-flight mass spectrometry (GCxGC-TOF-MS) [37], nanoflow liquid chromatography-electrospray ionization-tandem mass spectrometry (LC-ESI-MS/MS) [33] and liquid chromatography coupled with electrospray quadruple time-of-flight mass spectrometry (LC/QTOF-MS) [36]. A combination of two or more profiling methods has also been used by many research groups to help improve metabolite coverage [34], [40].

A comprehensive list of methods used to analyze urine and the numbers of metabolites identified and/or quantified by these methods (along with references) is provided in Table 1. As seen from this table, it is possible to (tentatively) identify up to 294 different metabolites in human urine. However, quantification is somewhat more difficult, with the largest number of quantified metabolites ever reported in human urine being slightly less than 100. In addition to these global metabolomic studies, hundreds of other “targeted” or single-metabolite studies have been conducted on human urine that have led to the identification and quantification of hundreds of other urine metabolites. Unfortunately, this information is not located in any central repository. Instead it is highly dispersed across numerous textbooks and periodicals [16].

**Table 1:** Historic data on metabolite characterization of human urine by different platforms.

Method(s)	# Identified	# Quantified	Year	Reference
Multiple	51	51	1971	Putnam [105]
GC-MS	52	0	1976	Lawson [85]
GC-MS	72	72	1978	Gates [106]
GC-MS	36	36	1989	Hoffman [107]
GC-MS	137	0	1990	Liebich [108]
GC-MS	131	95	1991	Shoemaker [84]
GC-MS	69	69	1994	Guneral [56]
GC-MS	75	75	1996	Matsumoto [109]
ICP-MS	33	33	2005	Goulle [110]
SPE/NMR	73	0	2008	Yang [40]
SPE/LC/NMR	72	0	2008	Rezzi [78]
LC-MS/NMR	50	0	2008	Law [34]
LC-MS	93	93	2009	Guo [111]
NMR	50	50	2009	Shaykhutdinov [112]
GC-MS-MS	48	48	2011	Kvitvang [113]
SPE-GC-MS	79	0	2011	Silva [114]
LC-MS	167	0	2011	Guo [115]
NMR	42	42	2011	Saude [116]
NMR/GC-MS	77	0	2012	Zhang [117]
LC-MS	219 (25 listed)	0	2012	Van der Kloet [118]
GC-TOF/LC-MS	261	0	2012	Cheng [119]
GC-MS	258	0	2012	Roux [83]
GCxGC-TOF-MS	294	0	2012	Rocha [120]

To facilitate future research into urine chemistry and urine metabolomics, we believe it is critical to establish a comprehensive, electronically accessible database of the detectable metabolites in human urine. This document describes just such a database, one that contains the metabolites that can, with today’s technology, be detected in human urine along with their respective concentrations and disease associations. This resource was assembled using a combination of both our own experimental and literature-based research. Experimentally, we used high-resolution NMR spectroscopy, gas chromatography mass spectrometry (GC-MS), direct flow injection tandem mass spectrometry (DFI/LC-MS/MS), inductively coupled plasma mass spectrometry (ICP-MS) and high performance liquid chromatography (HPLC) with ultraviolet (UV) or fluorescence detection (FD) techniques performed on multiple human urine samples to identify 445 metabolites or metabolite species and quantify 378 of these compounds. To complement these “global” metabolic profiling efforts, our team also surveyed and extracted metabolite and disease-association data from more than 3000 books and journal articles that had been identified through computer-aided literature and in-house developed text-mining software. This “bibliomic” effort yielded data for another 2206 metabolites. The resulting Urine Metabolome Database (UMDB - http://www.urinemetabolome.ca) is a comprehensive, web-accessible resource containing a total of 2651 confirmed urine metabolites or metabolite species (corresponding to 3079 defined structures), their corresponding concentrations and their disease associations that were revealed or identified from these combined experimental and literature mining efforts.

## Results and Discussion

### The Content of the Human Urine Metabolome – The Urine Metabolome Database

The Urine Metabolome Database (UMDB: http://www.urinemetabolome.ca) contains a complete list of all (to the best of our knowledge) possible metabolites that have been detected in human urine using current technologies. The UMDB is freely available, easily queried, web-enabled database which provides a list of the metabolite names, level of verification (confirmed or probable), normal and disease-associated concentration ranges, associated diseases and corresponding literature references for all human urine metabolites that have ever been detected and/or quantified in the literature. The UMDB also contains concentration data compiled from the experimental studies described here. Each urine metabolite entry in this database is linked to a MetaboCard button [15], [16] that, when clicked, brings up detailed information about that particular entry. This detailed information includes nomenclature, chemical, clinical and molecular/biochemical data. Each MetaboCard entry contains up to 120 data fields many of which are hyperlinked to other databases (KEGG [45], PubChem [46], MetaCyc [47], ChEBI [48], PDB [49], UniProt [50], and GenBank [51] as well as to GeneCard IDs [52], GeneAtlas IDs [53] and HGNC IDs [54] for each of the corresponding enzymes or proteins known to act on that metabolite). Additionally, the UMDB through its MetaboCard/HMDB links includes nearly 450 hand-drawn, zoomable and fully hyperlinked human metabolic pathway maps (SMPDB: http://www.smpdb.ca/). These maps are intended to help users visualize the chemical structures on metabolic pathways and to get detailed information about metabolic processes [55]. These UMDB pathway maps are quite specific to human metabolism and explicitly show the subcellular compartments where specific reactions are known to take place.

The UMDB’s simple text query (TextQuery) supports general text queries including names, synonyms, conditions and disorders. Clicking on the Browse button (on the UMDB navigation panel) generates a tabular view that allows users to casually scroll through the database or re-sort its contents by compound name or by concentration. Users can choose either the “Metabolite View”, “Concentration View” or “Diseases View” to facilitate their browsing or searching. Clicking on a given MetaboCard button brings up the full data content (from the HMDB) for the corresponding metabolite. Users may also search the database using a variety of options listed uner the “Search” menu. For instance, the ChemQuery button allows users to draw or write (using a SMILES string) a chemical compound to search the UMDB for chemicals similar or identical to the query compound. ChemQuery also supports chemical formula and molecular weight searches. The Sequence Search button allows users to conduct BLAST sequence searches of the 4075 protein sequences contained in the UMDB. Both single and multiple sequence BLAST queries are supported. “Advanced Search” which is also located under the “Search” menu is the most sophisticated search tool in the UMDB and opens an easy-to-use query search tool that allows users to select or search over various combinations of subfields. The UMDB’s “MS Search” allows users to submit mass spectral peak lists that will be searched against the Human Metabolome Database (HMDB)’s library of MS/MS spectra. This potentially allows facile identification of urine metabolites from mixtures via MS/MS spectroscopy. UMDB’s NMR Search allows users to submit peak lists from ^1^H or ^13^C NMR spectra (both pure and mixtures) and have these peak lists compared to the NMR libraries contained in the HMDB. This allows the identification of metabolites from mixtures via NMR spectral data. The Download button provides links to collected sequence, image and text files associated with the UMDB. In the About menu, the “Data Fields Explained” button lists source data used to assemble the UMDB.

Currently the UMDB contains information on 2651 *detectable* metabolites or metabolite species (which corresponds to 3079 metabolites with precisely defined structures) and 3832 concentration ranges or values associated with 220 different conditions, diseases and disorders. The number of metabolites in the UMDB is not a number that will remain unchanged. Rather it reflects the total number of metabolites – most of which are endogenous - that have ever been detected and/or quantified by ourselves and others. Certainly as technology improves, we anticipate this number will increase as other, lower abundance, metabolites are detected and added to future versions of the UMDB. Likewise, if the list was expanded to include intermittent, exogenous compounds such as all possible drugs or drug metabolites or rare food additives and food-derived phytochemicals, the database could be substantially larger.

Inspection of the on-line tables in UMDB generally shows that human urine contains a substantial number of hydrophilic molecules. This is further reiterated in Table 2, which provides a listing of the chemical “superclasses” (using the HMDB definitions) in human urine and the number of representative compounds that can be found in this biofluid. Excluding lipids (which are in very low concentration), human urine is dominated by amino acids and derivatives, carbohydrates and carbohydrate conjugates. This simply reinforces the fact that urine is a key carrier of hydrophilic waste products. Other small molecule components found in high abundance in urine include hydroxy acids and derivatives (such as citric acid), urea, ammonia, creatinine and hippuric acid. A more detailed description of both our literature and experimental findings is given in the following 7 sections covering: 1) Literature Review/Text Mining; 2) NMR; 3) DFI/LC-MS/MS; 4) GC-MS; 5) ICP-MS; 6) HPLC/UV and 7) HPLC/FD.

**Table 2:** Chemical superclasses in the urine metabolome database.

Chemical superclass	# Compounds
Aliphatic Acyclic Compounds	93
Aliphatic Heteromonocyclic Compounds	43
Aliphatic Heteropolycyclic Compounds	40
Aliphatic Homomonocyclic Compounds	18
Aliphatic Homopolycyclic Compounds	5
Alkaloids and Derivatives	45
Amino Acids, Peptides, and Analogues	286
Aromatic Heteromonocyclic Compounds	67
Aromatic Heteropolycyclic Compounds	728
Aromatic Homomonocyclic Compounds	432
Aromatic Homopolycyclic Compounds	6
Carbohydrates and Carbohydrate Conjugates	116
Homogeneous Metal Compounds	45
Homogeneous Non-metal Compounds	15
Inorganic compounds	1
Lignans and Norlignans	12
Lipids	866
Mixed Metal/Non-metal Compounds	7
Nucleosides, Nucleotides, and Analogues	49
Organic Acids and Derivatives	108
Organic Halides	3
Organometallic Compounds	1
Organophosphorus Compounds	17
Polyketides	74
Tannins	2

### Metabolite Concentration in Urine – Literature Survey

In addition to the experimentally derived values obtained for this study, the urine metabolome database (UMDB) also presents literature-derived concentrations of urine metabolites with references to either PubMed IDs or clinical textbooks. In many cases, multiple concentration values are given for “normal” conditions. This is done to provide users/readers with a better estimate of the potential concentration variations that different technologies or laboratories may measure. As a general rule, there is good agreement between most laboratories and methods. However, the literature results presented in the UMDB do not reflect the true state of the raw literature. A number of literature-derived concentration values were eliminated through the curation process after being deemed mistaken, disproven (by subsequent published studies), mis-typed or physiologically impossible. Much of the curation process involved having multiple curators carefully reading and re-reading the primary literature to check for unit type, unit conversion and typographical inconsistencies.

Other than the inorganic ions and gases such as sodium (14.7 ± 9.0 mM/mM creatinine – average value), chlorine (8.8 ± 6.2 mM/mM creatinine), potassium (4.6 ± 0.1 mM/mM creatinine), ammonia (2.8 ± 0.9 mM/mM creatinine), the 4 most abundant organic metabolites found in urine (based on average values) are urea (22.5 ± 4.4 mM/mM creatinine), creatinine (10.4 ± 2.0 mM), hippuric acid (298.5 ± 276.8 µM/mM creatinine) and citric acid (280.6 ± 115.2 µM/mM creatinine). The least abundant (detectable) metabolites in urine include oxytocin (0.9 ± 0.1 pM/mM creatinine), angiotensin II (1.2 ± 0.2 pM/mM creatinine), 15-deoxy-d-12,14-PGJ2 (2.3 ± 1.0 pM/mM creatinine) and melatonin (3.3 ± 2.7 pM/mM creatinine). This shows that the current lower limit of detection for metabolites in urine is in the low pM/mM creatinine range and that the concentration range of analytes in urine spans nearly 11 orders of magnitude.

One point that is particularly interesting is the fact that the concentration (scaled to creatinine) of the average metabolite in normal urine varies by about ± 50%, with some metabolites varying by as much as ± 350% (such as normetanephrine (0.00085 ± 0.00317 µM/mM creatinine), pipecolic acid (0.03 ± 0.07 µM/mM creatinine), enterodiol (0.032 ± 0.072 µM/mM creatinine), tungsten (0.010 ± 0.022 µM/mM creatinine) and chlorogenic acid (0.0014 ± 0.0029 µM/mM creatinine). Therefore, drawing conclusions about potential disease biomarkers without properly taking into account this variation would be ill-advised. We believe that these relatively large metabolite concentration ranges are due to a number of factors, including age, gender, genetic background, diurnal variation, health status, activity level and diet [56], [57], [58], [59]. Indeed, some UMDB entries explicitly show such variations based on the populations (age, gender) from which these metabolite concentrations were derived. Clearly more study on the contributions to the observed variations in urine is warranted, although with thousands of metabolites to measure for dozens of conditions, these studies will obviously require significant technical and human resources.

### Experimental Quantification and Identification of Urine Metabolites – NMR

**Figure 1:**
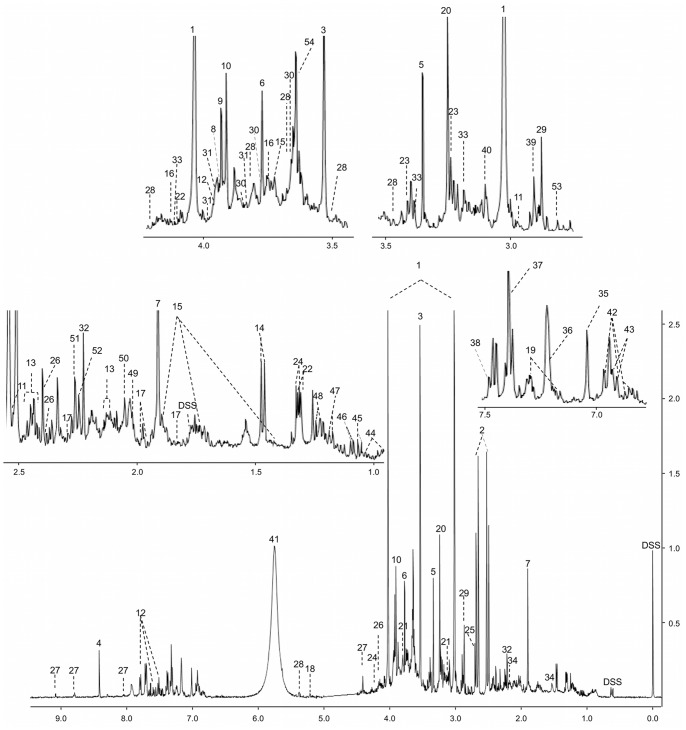
Typical 500 MHz ^1^H-NMR spectra of urine from human urine. Numbers indicates the following metabolites: 1: creatinine; 2: citric acid; 3: glycine; 4: formic acid; 5: methanol; 6: guanidoacetic acid; 7: acetic acid; 8: L-cysteine; 9: glycolic acid; 10: creatine; 11: isocitric acid; 12: hippuric acid; 13: L-glutamine; 14: L-alanine; 15: L-lysine; 16: gluconic acid; 17∶2- hydroxyglutaric acid; 18: D-glucose; 19: indoxyl sulfate; 20: trimethyl-N-oxide; 21: ethanolamine; 22: L-lactic acid; 23: taurine; 24: L-threonine; 25: dimethylamine; 26: pyroglutamic acid; 27: trigonelline; 28: sucrose; 29: trimethylamine; 30: mannitol; 31: L-serine; 32: acetone; 33: L-cystine; 34: adipic acid; 35: L-histidine; 36: L-tyrosine; 37: imidazole; 38: mandelic acid; 39: dimethylglycine; 40: Cis-aconitic acid; 41: urea; 42∶3-(3-hydroxyphenyl)-3-hydroxypropanoic acid (HPHPA); 43: phenol; 45: isobutyric acid; 46: methylsuccinic acid; 47∶3-aminoisobutyric acid; 48: L-fucose; 49: N-acetylaspartic acid; 50: N-acetylneuraminic acid; 51: acetoacetic acid; 52: Alpha-aminoadipic acid; 53: methylguanidine; 54: phenylacetylglutamine.

A representative high-resolution NMR spectrum of urine from a healthy individual is shown in Figure 1. As can be readily seen from this figure, urine NMR spectra are very information-rich and surprisingly complex, with thousands of resolved peaks. From the 22 healthy control urine samples analyzed, we could identify a total of 209 unique compounds with an average of 167 ± 19 compounds being identified per sample. Every compound was unequivocally identified and quantified using spectral fitting (via Chenomx) and/or spike-in experiments with authentic standards. The concentration of each metabolite was normalized to each urine sample’s corresponding creatinine value to compensate for variations in urine volume (the concentration of metabolites is expressed as µM/mM creatinine).

The 209 compounds identified and quantified from these NMR studies represent a “high-water” mark for NMR-based metabolomics. Previous studies have reported up to 70 compounds being identified and/or quantified in human urine [40]. Indeed, compared to other platforms previously used to analyze human urine (Table 1), it appears that NMR may currently be the most comprehensive and certainly the most quantitative approach to characterizing this biofluid. Based on the fitted area under each urinary NMR spectrum and the number of unidentified peaks we estimate that more than 96% of the spectral area and more than 92% of all NMR-detectable compounds in our human urine samples are listed in Table 3. In other words, for NMR-based metabolomics, human urine is essentially “solved”. The same “solved” status has already been achieved for human serum (with 49 definitive compounds, [20]) and for human cerebrospinal fluid (with 53 definitive compounds, [17]). Knowing the expected or detectable composition of these biofluids should open the door to automated NMR-based metabolomics [60].

**Table 3:** Concentrations [Mean (range)] and % occurrence of urine metabolites as determined by NMR.

Compounds	HMDB ID	Concentration (µM/mM creatinine)	Oc (%)	Literature Values (µM/mM creatinine)
1,3-Diaminopropane	HMDB00002	1.2	5	2.55 (0.18–5.85)
1,3-Dimethyluric acid	HMDB01857	3.1 (1.3–6.8)	68	1.5 (1.3–2.3)
1-Methyladenosine	HMDB03331	1.7 (0.9–3.6)	82	2.03±0.20
1-Methylhistidine	HMDB00001	8.3 (2.4–28.4)	100	15.9±19.5
1-Methylnicotinamide	HMDB00699	5.8 (1.2–15.0)	77	6.1 (0.2–12.0)
2-Furoylglycine	HMDB00439	4.0 (0.9–8.4)	100	9.95 (2.0–18.66)
2-Hydroxy-3-methylpentanoic acid	HMDB00317	3.9 (1.7–7.9)	100	NA
2-Hydroxybutyric acid	HMDB00008	2.8 (1.2–6.9)	77	2.4 (0.0–4.9)
2-Hydroxyglutaric acid	HMDB00694	33.0 (13.3–77.9)	100	33.0 (20.0–46.0)
2-Ketobutyric acid	HMDB00005	2.9 (0.7–5.4)	100	2.6 (1.1–4.1)
2-Methyl-3-hydroxybutyric acid	HMDB00354	4.2 (1.6–6.7)	95	5.5 (0.0–11.0)
2-Methyl-3-ketovaleric acid	HMDB00408	1.1 (0.8–1.3)	18	NA
2-Methylbutyrylglycine	HMDB00339	2.0 (1.2–3.3)	32	1 (0–2)
2-Methylerythritol	HMDB11659	25.3 (17.0–49.0)	14	NA
2-Methylglutaric acid	HMDB00422	0.8 (0.3–1.7)	45	NA**
3-Aminoisobutanoic acid	HMDB03911	26.0 (2.2–140.0)	100	2.91–116.43
3-Hydroxybutyric acid	HMDB00357	3.6 (1.3–6.4)	77	1.4±1.3
3-Hydroxyhippuric acid	HMDB06116	7.4 (5.7–9.1)	32	2.5 (0.2–5.0)
3-Hydroxyisovaleric acid	HMDB00754	6.8 (3.2–21.8)	100	8.5±3.2
3-Hydroxymethylglutaric acid	HMDB00355	2.8 (1.1–5.8)	68	3 (0–10)
3-Hydroxyphenylacetic acid	HMDB00440	7.7 (1.4–22.1)	91	6.1±4.1
3-Methyl-2-oxovaleric acid	HMDB00491	2.2 (0.4–4.8)	91	2.0±2.0
3-Methyladipic acid	HMDB00555	2.9 (1.1–7.2)	86	2.9 (0.7–10.5)
3-Methylglutaconic acid	HMDB00522	7.5 (3.4–9.5)	22	4.5 (0.0–9.0)
3-Methylhistidine	HMDB00479	16.5 (2.8–59.8)	100	15.1 (3.9–26.3)
3-Methylxanthine	HMDB01886	6.2 (1.4–17.8)	100	4.0 (2.3–6.3)
4-Aminobutyric acid	HMDB00112	2.9 (2.7–3.3)	9	0.75 (0.0–1.50)
4-Aminohippuric acid	HMDB01867	3.3 (1.0–7.2)	86	0.3
4-Deoxythreonic acid	HMDB02453	12.3 (4.4–19.8)	100	25±10
4-Ethylphenol	HMDB29306	0.9 (0.6–1.2)	18	0.409±0.174
4-Hydroxybenzoic acid	HMDB00500	1.7 (0.9–3.0)	77	1.8±1.8
4-Hydroxybutyric acid	HMDB00710	7.2 (2.7–22.3)	100	3.3 (0.0–10.8)
4-Hydroxyhippuric acid	HMDB13678	2.8 (1.4–6.0)	100	0–14
4-Hydroxyphenyllactic acid	HMDB00755	3.2 (1.5–3.9)	50	1.1 (0.2–2.6)
4-Pyridoxic acid	HMDB00017	3.1 (0.4–7.5)	95	0.78±0.45a
5,6-Dihydrouracil	HMDB00076	3.8 (2.1–8.3)	68	3.0±2.7
5-Aminolevulinic acid	HMDB01149	2.9 (1.2–4.4)	95	1.45±0.72
5-Aminopentanoic acid	HMDB03355	1.1	5	2
7-Methylxanthine	HMDB01991	10.0 (3.9–16.1)	9	10.0 (6.7–14.6)
Acetaldehyde	HMDB00990	2.6 (0.8–4.2)	32	NA**
Acetaminophen	HMDB01859	11.1	5	5.3
Acetaminophen glucuronide	HMDB10316	33.4	5	NA**
Acetaminophen sulfate	HMDB59911	138.6	5	NA**
Acetic acid	HMDB00042	13.0 (2.5–106.0)	100	45.3±53.3
Acetoacetic acid	HMDB00060	11.1 (2.2–24.9)	100	33 (0–67)
Acetone	HMDB01659	3.9 (0.8–17.6)	100	2.24±4.13
Acetyl-L-carnitine	HMDB00201	2.8 (0.6–7.5)	100	1.9±0.3
Adenine	HMDB00034	2.9 (1.4–5.1)	45	1.17
Adenosine	HMDB00050	1.4 (0.9–2.3)	45	0.50 (0.09–0.92)
Adipic acid	HMDB00448	4.4 (1.6–14.3)	91	5.1 (0.8–35.0)
ADP	HMDB01341	1.8 (1.0–3.4)	77	NA**
Allantoin	HMDB00462	15.4 (4.9–29.3)	100	19.7±10.5
Alpha-Aminoadipic acid	HMDB00510	7.3 (3.4–11.2)	73	4.6
Alpha-Aminobutyric acid	HMDB00452	2.2 (1.1–4.4)	95	1.37±1.05
Alpha-Hydroxyisobutyric acid	HMDB00729	3.8 (1.7–5.9)	100	3.8
Alpha-Ketoisovaleric acid	HMDB00019	0.5 (0.2–1.1)	50	1 (0–2)
Anserine	HMDB00194	7.0 (3.3–20.0)	100	16.16
Arabinitol	HMDB01851	31.8 (10.2–64.3)	100	19.28±6.32
Ascorbic acid	HMDB00044	32.5 (4.6–78.0)	14	1.70–22.72
Asymmetric dimethylarginine	HMDB01539	3.0 (0.2–6.7)	100	4.6 (3.0–7.0)
Azelaic acid	HMDB00784	2.8 (1.8–4.8)	64	4.8 (1.3–15.0)
Benzoic acid	HMDB01870	4.7 (1.2–10.5)	86	4.2 (1.9–6.5)
Beta-Alanine	HMDB00056	5.9 (3.4–13.0)	68	5 (0–10)
Betaine	HMDB00043	11.5 (2.7–24.7)	100	4.14
Butyric acid	HMDB00039	1.6 (0.5–4.0)	77	0.131±0.013
Caffeine	HMDB01847	1.2	5	0.33 (0.0–1.01)
Carnosine	HMDB00033	5.5 (1.4–12.6)	91	4.0±2.5
Choline	HMDB00097	3.5 (1.4–6.1)	100	5.27
Cinnamic acid	HMDB00567	1.6 (0.9–2.2)	9	0.77
Cis-Aconitic acid	HMDB00072	20.9 (3.8–95.3)	100	13.0 (2.7–44.0)
Citraconic acid	HMDB00634	2.0 (1.2–2.6)	73	2.2
Citramalic acid	HMDB00426	2.4 (1.0–4.8)	91	3.1
Citric acid	HMDB00094	203 (49–600)	100	242.0±129.6
Creatine	HMDB00064	46 (3–448)	100	46 (9–135)
Creatinine*	HMDB00562	14,743±9,797	100	10,090±310
Cytosine	HMDB00630	4.1 (1.1–10.7)	36	NA**
Dehydroascorbic acid	HMDB01264	9.1 (5.8–7.1)	32	1.72–7.47
D-Galactose	HMDB00143	11.9 (5.4–25.2)	100	4.4 (0.0–31.0)
D-Glucose	HMDB00122	37.5 (12.5–58.4)	100	31.12
Dihydrothymine	HMDB00079	2.4 (1.3–4.4)	64	5 (0–10)
Dimethyl sulfone	HMDB04983	8.0 (1.3–49.0)	100	4.6 (2.5–7.5)
Dimethylamine	HMDB00087	30.8 (20.3–59.2)	100	39.3±8.3
Dimethylglycine	HMDB00092	4.4 (1.6–10.4)	100	6.2 (0.8–11.2)
D-Threitol	HMDB04136	19.3 (5.3–32.7)	100	10.0 (5.6–15.0)
D-Xylitol	HMDB02917	8.4 (6.0–13.8)	95	3.50±2.37
D-Xylose	HMDB00098	20 (3.0–102)	91	51.19
Erythritol	HMDB02994	33.4 (6.8–64.0)	100	51±18
Ethanol	HMDB00108	3.1	5	5–500^b^
Ethanolamine	HMDB00149	37.0 (24.8–56.2)	100	21.4 (6.6–36.2)
Ethyl glucuronide	HMDB10325	8.2	5	26.38±15.26
Ethylmalonic acid	HMDB00622	3.0 (1.2–5.8)	95	2.0 (0.5–3.9)
Formic acid	HMDB00142	26.8 (6.9–120.9)	100	20.39±11.84
Fumaric acid	HMDB00134	0.7 (0.2–1.7)	50	1 (0–2)
Glucaric acid	HMDB00663	8.4 (5.8–12.3)	82	2.9±0.2
Gluconic acid	HMDB00625	21.5 (8.1–38.8)	100	NA**
Glucuronic acid	HMDB00127	9.7 (3.7–20.6)	77	NA
Glutaconic acid	HMDB00620	3.1 (1.2–3.1)	68	1 (0–2)
Glutaric acid	HMDB00661	2.1 (0.7–3.6)	18	1.3 (0.6–2.6)
Glyceric acid	HMDB00139	15.9 (7.8–31.1)	100	4.5 (0.0–9.0)
Glycerol	HMDB00131	13 (4–19)	100	20 (0–40)
Glycine	HMDB00123	106 (44–300)	100	151 (233–248)
Glycolic acid	HMDB00115	42.0 (3.7–122.0)	100	35 (18–55)
Guanidoacetic acid	HMDB00128	41.8 (10.6–97.3)	100	89.0 (11.0–124.0)
Hippuric acid	HMDB00714	229 (19–622)	100	257 (20–770)
Homocitrulline	HMDB00679	7.5 (3.8–13.8)	91	6.0 (0.0–8.8)
Homogentisic acid	HMDB00130	1.70 (0.55–2.8)	45	1 (0–2)
Homovanillic acid	HMDB00118	6.2 (1.8–12.7)	100	4.9±3.4
HPHPA	HMDB02643	5.9 (1.4–22.1)	100	0.55–54.93
Hydroxyproline	HMDB00725	6.5 (2.4–12.5)	55	2
Hydroxypropionic acid	HMDB00700	12.3 (4.4–17.9)	82	6.5 (3.0–10.0)
Hypoxanthine	HMDB00157	7.2 (1.8–24.1)	100	4.67 (2.80–6.38)
Indoleacetic acid	HMDB00197	3.4 (1.8–6.2)	100	2.0±1.0
Indoxyl sulfate	HMDB00682	22.4 (6.0–64.8)	100	19.74±5.26
Inosine	HMDB00195	1.4 (0.4–6.5)	50	1.04±0.56
Isobutyric acid	HMDB01873	3.5 (1.2–9.0)	95	3.9
Isocitric acid	HMDB00193	46.9 (20.4–89.2)	68	58 (36–84)
Isovaleric acid	HMDB00718	1.4 (0.8–2.5)	86	0.42±0.02
Isovalerylglycine	HMDB00678	2.0 (0.4–4.0)	91	5.5 (1.0–10.0)
Kynurenic acid	HMDB00715	2.1 (0.8–4.2)	64	1.53 (0.66–4.13)
Kynurenine	HMDB00684	1.7 (1.1–2.5)	23	2.85±1.94
Lactose	HMDB00186	11.8 (1.0–24.2)	91	36.31±6.6^c^
L-Alanine	HMDB00161	21.8 (7.1–43.0)	100	22.0±11.0
L-Arabinose	HMDB00646	13.3 (1.3–22.0)	100	8.75 (6.12–11.84)
L-Arginine	HMDB00517	8.6 (3.8–18.6)	95	6.6 (2.5–10.8)
L-Asparagine	HMDB00168	10.0 (4.6–17.8)	95	10.0 (4.6–16.3)
L-Aspartic acid	HMDB00191	10.9 (3.5–21.8)	95	4.63 (1.71–7.56)
L-Carnitine	HMDB00062	5.0 (0.7–16.4)	100	5.7±4.8
L-Cystathionine	HMDB00099	14.7 (3.9–20.7)	82	5.3 (1.0–9.6)
L-Cysteine	HMDB00574	65.8 (23.1–134.5)	100	33.4±15.5
L-Cystine	HMDB00192	12.5 (5.0–24.6)	100	9.0±3.0
Levoglucosan	HMDB00640	10.0 (2.4–29.0)	27	NA**
Levulinic acid	HMDB00720	1.3 (0.4–2.0)	64	1.28 (0.33–2.75)
L-Fucose	HMDB00174	11.8 (6.7–26.0)	91	9.6 (6.4–16.0)
L-Glutamic acid	HMDB00148	8.5 (3.3–18.4)	100	10.0±3.7
L-Glutamine	HMDB00641	37.2 (19.1–77.9)	100	33.32±10.78
L-Histidine	HMDB00177	43 (17–90)	100	60.75±27.45
L-Isoleucine	HMDB00172	1.3 (0.4–2.6)	100	1.64
L-Lactic acid	HMDB00190	11.6 (3.5–29.3)	100	12.3±6.2
L-Leucine	HMDB00687	2.8 (1.6–5.4)	100	2.5±1.3
L-Lysine	HMDB00182	17.2 (3.7–51.3)	100	18 (7–29)
L-Methionine	HMDB00696	1.4 (0.5–2.5)	41	1.3±0.97
L-Phenylalanine	HMDB00159	6.4 (3.5–11.2)	100	4.50±1.15
L-Serine	HMDB00187	25.3 (11.6–53.4)	100	26.0±10.0
L-Threonine	HMDB00167	13.3 (6.4–25.2)	100	5.17–24.59
L-Tryptophan	HMDB00929	6.3 (3.4–11.1)	100	2.45–29.40
L-Tyrosine	HMDB00158	9.5 (4.1–23.5)	100	8.8±4.5
L-Valine	HMDB00883	3.4 (2.0–7.7)	100	3.0±1.0
Maleic acid	HMDB00176	0.4 (0.3–0.5)	14	0.5
Malonic acid	HMDB00691	2.9 (2.0–3.5)	77	1 (0–2)
Maltose	HMDB00163	6.0 (1.3–21.4)	73	NA**
Mandelic acid	HMDB00703	1.4 (1.1–1.7)	9	0.966±0.669
Mannitol	HMDB00765	32.4 (5.2–85.1)	95	10.26±9.14
Methanol	HMDB01875	37 (10–117)	100	NA**
Methylamine	HMDB00164	4.0 (1.5–11.9)	100	10.5 (9.8–12.5)
Methylglutaric acid	HMDB00752	3.1 (2.6–3.6)	9	3.5 (0.0–7.0)
Methylguanidine	HMDB01522	2.7 (1.2–6.0)	95	1.25±0.72
Methylmalonic acid	HMDB00202	1.9 (0.7–3.5)	95	1.6 (0.0–3.6)
Methylsuccinic acid	HMDB01844	3.2 (0.7–9.9)	100	2.3 (0.8–10.8)
Monomethyl glutaric acid	HMDB00858	2.3 (0.6–4.4)	68	NA
m-Thymol	HMDB01878	2.2 (0.7–4.0)	86	NA**
Myoinositol	HMDB00211	22.4 (7.9–36.1)	100	18.8±2.9
N-Acetylaspartic acid	HMDB00812	4.0 (1.3–7.0)	100	4.66±1.14
N-Acetylneuraminic acid	HMDB00230	5.4 (2.5–8.6)	100	8.60±1.58
N-Acetylputrescine	HMDB02064	0.5 (0.4–0.7)	32	1.18 (0.58–2.25)
N-Methylhydantoin	HMDB03646	4.9 (1.0–11.0)	55	NA
o-Hydroxyphenylacetic acid	HMDB00669	2.0 (0.9–4.5)	91	7.71±3.65^d^
Oxoglutaric acid	HMDB00208	4.8 (2.0–17.0)	100	2.98 (0.42–18.30)
Oxypurinol	HMDB00786	13.3 (5.1–29.3)	50	3.29
Pantothenic acid	HMDB00210	1.9 (0.6–4.4)	100	2.7±0.9
p-Cresol sulfate	HMDB11635	1.3 (0.3–5.5)	91	NA**
Phenol	HMDB00228	4.8 (0.6–12.8)	95	7.0 (5.6–9.2)
Phenylacetic acid	HMDB00209	1.9	5	4.16±0.91
Phenylacetylglutamine	HMDB06344	34.0 (4.5–70.0)	100	47.03 (3.84–85.51)
Phenylglyoxylic acid	HMDB01587	7.34	5	36.6±0.48^e^
Phosphorylcholine	HMDB01565	1.1 (0.7–3.0)	41	NA
p-Hydroxyphenylacetic acid	HMDB00020	5.5 (1.4–14.6)	100	9.68
Picolinic acid	HMDB02243	19.4 (7.2–37.6)	18	1.65–10.28
Pimelic acid	HMDB00857	2.2 (0.7–4.0)	77	NA**
Proline-betaine	HMDB04827	2.3 (0.8–4.0)	100	12.7 (1.6–23.7)
Propylene glycol	HMDB01881	6.7 (1.4–44.3)	100	NA**
Pseudouridine	HMDB00767	28.9 (13.3–41.3)	100	26.02±4.62
Pyrocatechol	HMDB00957	4.7 (2.0–8.5)	100	6.0±4.3
Pyroglutamic acid	HMDB00267	20.7 (10.2–32.6)	100	28.8 (3.4–54.2)
Pyruvic acid	HMDB00243	2.7 (0.8–6.4)	100	2.13 (0.54–8.67)
Quinolinic acid	HMDB00232	5.2 (1.0–17.5)	100	2.5±1.1
Sarcosine	HMDB00271	2.9 (0.5–5.4)	77	2.8 (0.0–5.6)
Scyllitol	HMDB06088	4.2 (2.2–8.1)	68	NA
Sebacic acid	HMDB00792	1.9	5	1 (0–2)
Sorbitol	HMDB00247	9.9 (2.5–18.7)	100	3.50±2.24
Suberic acid	HMDB00893	1.6 (0.9–2.4)	32	0.5 (0.0–2.9)
Succinic acid	HMDB00254	6.0 (0.3–33.3)	91	5.6±3.8
Succinylacetone	HMDB00635	2.8 (0.6–4.7)	36	1 (0–2)
Sucrose	HMDB00258	7.4 (1.4–19.5)	100	7.12±3.23
Sumiki’s acid	HMDB02432	1.7	5	NA**
Symmetric dimethylarginine	HMDB03334	2.7 (1.5–5.2)	100	3.72±0.35
Syringic acid	HMDB02085	1.5	5	0.9±0.2
Tartaric acid	HMDB00956	11.8 (2.6–64.4)	82	12.0 (1.3–46.0)
Taurine	HMDB00251	81 (13–251)	100	4.00–159.98
Threonic acid	HMDB00943	20.8 (10.2–39.3)	100	10.0±7.0
Thymidine	HMDB00273	2.2 (0.7–3.9)	36	6.87±4.86
Trans-Aconitic acid	HMDB00958	6.6 (1.8–25.1)	55	3.38
Trans-Ferulic acid	HMDB00954	1.2	5	36.3±11.2^f^
Trigonelline	HMDB00875	31.1 (5.5–109.3)	100	16.08
Trimethylamine	HMDB00906	2.5 (0.3–19.4)	86	7.7±7.4
Trimethylamine N-oxide	HMDB00925	91.0 (4.8–509.0)	100	118.7 (35.5–202.1)
Uracil	HMDB00300	9.5 (2.6–22.8)	100	12 (2–22)
Urea	HMDB00294	12,285 (174–49,097)	100	22,566±4,407
Vanillic acid	HMDB00484	1.6 (0.6–3.3)	95	1.0 (0.0–2.5)
Vanillylmandelic acid	HMDB00291	2.3 (1.0–3.4)	9	1.2±0.5

* Concentration of metabolite (Mean ± SD) is expressed by µM.

a The mean daily vitamin B6 intake was well below the recommended dietary allowance for men and woman;

b depends greatly on amount of alcohol consumed;

c after administration of lactose;

d after administration of 1 g of coumarin;

e After styrene exposure; and

f after consumption of chocolate.

NA: not available; NA**: not available for healthy adults; ADP: Adenosine-5-diphosphate; HPHPA: 3-(3-hydroxyphenyl)-3-hydroxypropanoic acid; Oc: occurrence.

Based on these NMR studies, the most abundant constituents (based on average values) of urine from a healthy individual are urea (12.3 ± 14.5 mM/mM creatinine), creatinine (14.7 ± 9.8 mM), hippuric acid (229 ± 160 µM/mM creatinine) and citric acid (203 ± 129 µM/mM creatinine). The least abundant compounds were trans-ferulic acid (1.2 µM/mM creatinine), phosphorylcholine (1.1 ± 0.7 µM/mM creatinine), 4-ethylphenol (0.9 ± 0.1 µM/mM creatinine), 2-methylglutaric acid (0.8 ± 0.4 µM/mM creatinine). The lowest concentrations that could be reliably detected using NMR were 0.4 ± 0.1 µM/mM creatinine (for maleic acid), 0.5 ± 0.2 µM/mM creatinine (for alpha-ketoisovaleric acid and N-acetylputrescine) and 0.7 ± 0.4 µM/mM creatinine (for fumaric acid). The complete list of average compound concentrations (including their range), literature-reported concentrations (for healthy adult) and the frequency of their occurrence is shown in Table 3. In general, there is good agreement between the NMR-measured concentrations and those reported in the literature (often obtained by other analytical techniques).

However, not all of the NMR-derived urine concentrations agree with literature-derived values. A total of 34 compounds had average concentrations somewhat higher (>1 SD) than previously reported values (for example, 1, 3-dimethyluric acid, glucaric acid, L-aspartic acid, mannitol), while 21 compounds had average concentrations lower (<1 SD) than previously reported (for example, 1-methylhistidine, phenol, dihydrothymine, thymidine). Another 7 metabolites also exhibited somewhat greater range in concentrations than those previously reported in the literature. These included: acetic acid, butyric acid, isovaleric acid, lactose, phenylglyoxylic acid, proline-betaine and trans-ferulic acid. Some of these discrepancies are likely due to differences in diet, physiological status, pharmacological effects and the age of the different cohorts that were analyzed. Other differences may be due to storage effects, sample preparation methods and the analytical methods being used. As a rule, NMR concentration determinations are very accurate since they involve direct measurement of the compound, as opposed to an indirect measurement of a derivative compound. Therefore we are quite confident in the NMR concentration values reported in Table 3 and would tend to view these as more reliable than those measured via other technologies.

A number of the compounds exhibiting higher-than or lower-than-reported concentrations appear to be associated with dietary intake. For example, mannitol is a sugar alcohol that is poorly absorbed by humans but its presence in urine can be explained by its occurrence in commonly consumed foods such as apples, pineapples, asparagus and carrots. Likewise, the urinary excretion of trans-ferulic acid (a polyphenolic derivative) increases after the ingestion of breakfast cereals [61] and chocolate. The relatively low value we measured is likely due to the fact that the literature value of trans-ferulic acid reported in Table 3 was measured for people on a special diet [62]. Also, the low level of proline-betaine we measured in urine may be due to a lower frequency of exposure to dietary citrus fruits in our population sample [63]. Proline-betaine is an osmoprotectant found in citrus fruit and urinary excretion of this metabolite is increased after consumption of fruits such as orange juice [64]. Similarly the higher levels of dimethyl sulfone we detected in urine could be attributed to dietary sources that contain DMSO [65]. For example, onions contain many sulfoxides including DMSO which can be oxidized in the liver and kidneys to produce dimethyl sulfone [66], [67]. The consumption of meat could also significantly increase the concentration of some metabolites in urine as reported in the case of 1-methylhistidine [68]. 1-methylhistidine is produced from the metabolism of anserine (a dipeptide) which is commonly found in meats [69], [70]. In addition to diet, metabolite levels in urine can also be affected by physiological status. For instance, the level of 3-hydroxybutyric acid in urine increases during fasting and can range from 0 to 200 µM/mM creatinine, with the maximum level reported in the literature (200 µM/mM creatinine) corresponding to healthy male after 35 h of fasting [50].

Some of the metabolites we measured by NMR did not have any previously reported literature values. For example, glucuronic acid is usually reported as total glucuronic acid (the free acid plus glucuronide conjugates) after hydrolysis [71], [72]. Here we report the concentration of free glucuronic acid, as indicated in Table 3. Another example, 2-methylerythritol, was previously detected in human urine but no concentration was reported [73]. The urinary excretion of 2-methylerythritol is most likely a result of dietary consumption of fruits or vegetables containing 2-methylerythritol and/or 2-methylerythritol-4-phosphate. 2-methylerythritol-4-phosphate is an intermediate in isoprenoid biosynthesis [74] and has been found to be quite abundant in certain plants [75].

A number of compounds we measured by NMR appear to be normal constituents of human urine but seem not to have been previously reported as being detectable by NMR (a total of 42 compounds) or reported as detected but not-quantified by any other method (a total of 8 compounds). The identification of these “NMR-novel” compounds was aided by their prior identification by GC-MS and DI-MS (see following sections) and through a careful literature analysis of compounds that had previously been detected in human urine via other methods. The list of detected but not previously quantified by NMR compounds includes: 2-hydroxy-3-methylpentanoic acid, 2-methyl-3-ketovaleric acid, 2-methylerythritol, glucuronic acid, monomethyl glutaric acid, N-methylhydantoin, phosphorylcholine and scyllitol. All of these compounds were confirmed using authentic standards.

This NMR study also revealed a number of common identification errors made in previously published NMR-based human urine metabolomic studies. In particular, several earlier reports identified phenylacetylglycine [76], N-acetylglutamic acid [77], cresol [78], isonicotinic acid [78], yellow 7.1 [79], meta-hydroxyphenylpropionic acid [80], 2-oxoisocaproic acid [81], urocanic acid, glycylproline and ornithine [80] as being detectable by NMR in human urine. Using our NMR instrument and the samples available to us, we were unable to detect any of these compounds, even after performing multiple spike-in experiments using authentic compounds. While some of these metabolites have been previously reported to be in human urine, they were reported at concentrations far below the lower limit of detection of modern NMR instruments (which is ∼ 1 µM). Due to their chemical shift similarity, phenylacetylglycine (which is found only in rats and mice) and N-acetylglutamic acid appear to be commonly mistaken for phenylacetylglutamine. We also noticed that, isonicotinic acid (a breakdown product of isoniazid and hydrazine derivatives, which is found only in individuals that have taken isoniazid and other hydrazine derivatives as a drug) appears to be mistaken for trigonelline. Likewise cresol (water-insoluble) appears to be frequently mistaken for cresol-sulfate (water-soluble), while the compounds yellow 7.1, meta-hydroxyphenylpropionic acid and 3-(p-hydroxyphenyl)-propionic acid appear to be commonly mistaken for 3-(3-hydroxyphenyl)-3-hydroxypropanoic acid (HPHPA).

In addition to correcting these compound identification errors, we also observed some significant gender-related effects on creatinine levels in our urine samples. Since males generally have a greater mass of skeletal muscle than females, they tend to have higher urinary levels of creatinine than women. This was clearly evident in our samples as the average male creatinine level was 20 mM while the average female creatinine level was 11 mM. In addition, increased dietary intake of creatine or a protein-rich diet can increase daily creatinine excretion [82].

### Quantification and Identification of Urine Metabolites – GC-MS

**Figure 2:**
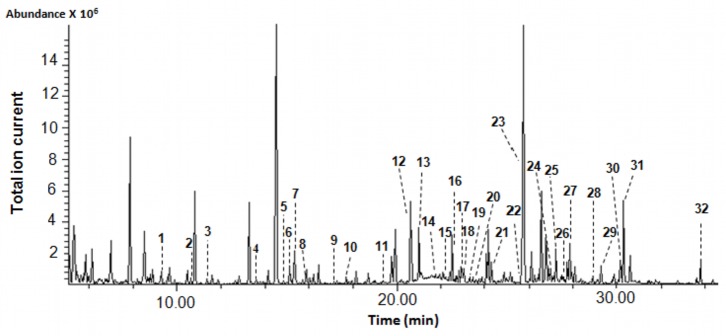
Typical total ion chromatogram of water-soluble metabolites extracted from human urine. Numbers indicate the following metabolites (quantified compounds): 1: L-valine; 2: oxalic acid; 3∶3-hydroxyisobutyric acid; 4: L-serine; 5: glycine; 6: succinic acid; 7: glyceric acid; 8∶4-deoxythreonic acid; 9∶2,4-dihydroxybutanoic acid; 10∶3,4-dihydroxybutanoic acid; 11: adipic acid; 12: creatinine; 13: threonic acid; 14: L-phenylalanine; 15: p-hydroxyphenylacetic acid; 16: L-ornithine; 17: L-asparagine; 18: L-arabinose; 19: D-xylitol; 20: D-xylulose; 21: Cis/Trans-aconitic acid; 22: hippuric acid; 23: isocitric acid; 24: D-galactose; 25: D-glucose; 26: L- tyrosine; 27: sorbitol; 28: gluconic acid; 29: scyllitol; 30: myoinositol; 31: uric acid; 32: pseudouridine.

**Figure 3:**
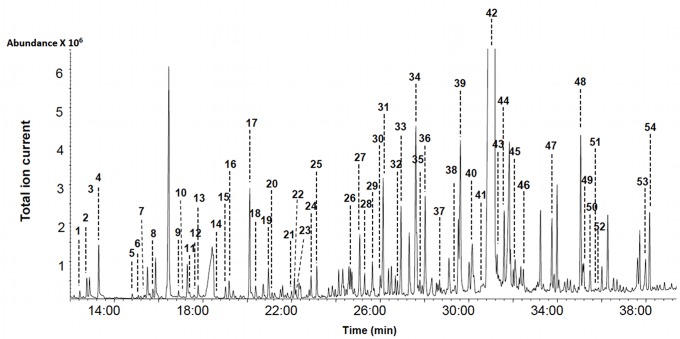
Typical GC-MS total ion chromatogram of organic acids extracted from human urine. Numbers indicate the following metabolites (quantified compounds): 1: pyruvic acid; 2: L-lactic acid; 3: alpha-hydroxyisobutyric acid; 4: glycolic acid; 5: levulinic acid; 6∶3-hydroxyisovaleric acid; 7∶2-hydroxy-2-methylbutyric acid; 8: hydroxypropionic acid; 9∶2-methyl-3-hydroxybutyric acid; 10: malonic acid; 11∶3-hydroxyisovaleric acid; 12: methylmalonic acid; 13∶2-ethylhydracrylic acid; 14: benzoic acid; 15: phosphoric acid; 16: ethylmalonic acid; 17: succinic acid; 18: methylsuccinic acid; 19∶4-deoxythreonic acid; 20∶5-hydroxyhexanoic acid; 21: citraconic acid; 22: glutaric acid; 23: m-chlorobenzoic acid; 24; 3,4-dihydroxybutanoic acid; 25∶3-methylglutaconic acid; 26: adipic acid; 27: pyroglutamic acid; 28∶3-methyladipic acid; 29: sumiki’s acid; 30: o-hydroxyphenylacetic acid; 31: oxoglutaric acid; 32: pimelic acid; 33∶3-hydroxymethylglutaric acid; 34∶3-hydroxyphenylacetic acid; 35∶4-hydroxybenzoic acid; 36∶2-furoylglycine; 37: suberic acid; 38: quinolinic acid; 39: Cis/Trans-aconitic acid; 40: homovanillic acid; 41: azelaic acid; 42: hippuric acid; 43∶3,4-dihydroxybenzeneacetic acid; 44∶3-(3-hydroxyphenyl)-3-hydroxypropanoic acid (HPHPA); 45: vanillylmandelic acid; 46∶4-hydroxyphenyllactic acid; 47: indoleacetic acid; 48: palmitic acid; 49: kynurenic acid; 50∶3-hydroxyhippuric acid; 51∶3-hydroxysebacic acid; 52: Trans-ferulic acid; 53∶5-hydroxyindoleacetic acid; 54: stearic acid.

**Figure 4:**
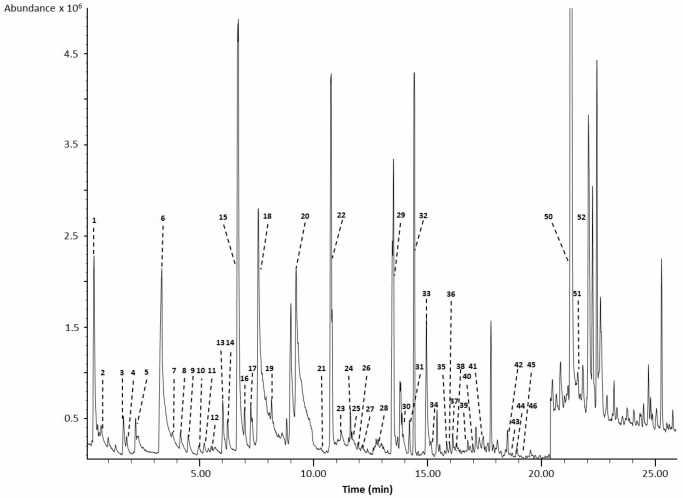
Typical gas chromatogram of volatile organic compounds (VOC) from a pooled human urine sample. Numbers indicate the following metabolites: 1∶3-methyl sulfolane; 2∶3-hexanone; 3∶2-pentanone; 4∶1-hydroxy-2-pentanone; 5: allyl methylsulphide; 6: dimethyl disulfide; 7∶4-heptanone; 8∶1-methylcyclohexanol; 9; 2-hexanone; 10∶3,4-dimethylthiophene; 11: diallyl sulphide; 12∶5-methyl-2-hexanone; 13∶1,3-dithio cyclohexane; 14: dimethyl trisulfide; 15: phenol; 16: o-cymene; 17: p-cymene; 18: m-cymene; 19∶1,4-cineol; 20: p-cresol; 21: linalool oxide; 22: iso-menthol; 23: Alpha-p-dimethylstyrene; 24: L-menthol; 25: undecane; 26: ledene oxide (II); 27: salicylic acid methyl ester; 28: Beta-carvone; 29: piperitone; 30: o-thymol; 31: Beta-cyclocitrol; 32∶4,7-dimethyl-benzofuran; 33: cuminal: 34∶2,6,10,10 Tetramethyl-oxa-spiro-4,5-dec-6-ene; 35∶4-(2,6,6-trimethyl-1-cyclohexen-1-yl)-2-butanone; 36∶1,2,3,4-tetrahydro-1,1,6-trimethyl naphthalene; 37: Alpha-cedrene; 38∶1,2,3,4-tetrahydro-1,5,7-trimethylnapthalene; 39∶1,2-diydro-1,1,6-trimethyl-napthalene; 40: Beta-guaiene; 41: Beta-damascenone; 42∶2,5-cyclohexadiene-1,4-dione-2,6-di-tert-butyl; 43: himachalene; 44∶4-(2,6,6-trimethylcyclohexa-1,3-dienyl)-but-3-en-2-one: 45∶1-(2,6,6-trimethyl-1-cyclohexen-1-yl)-1-penten-3-one; 46∶2,4-Bis (1,1-dimethylethyl)-phenol; 47∶1-(2,3,6-trimethyl phenyl)-3-buten-2-one; 48: L-calamenene; 49: Beta-vatirenene; 50∶1,6,7-trimethylnaphthalene; 51: azulol; 52∶3,3,5,6-tetramethyl-1-indanone.

As seen in Table 1, GC-MS methods have long been used to comprehensively characterize the chemical content of human urine. For our studies a total of 4 different GC-MS analyses were performed. The first method employed polar solvent extraction and derivatization to achieve broad metabolite coverage of polar metabolites, the second was more selective and targeted organic acids, the third targeted volatiles, while the fourth targeted bile acids. Representative high-resolution GC-MS total ion chromatograms are shown in Figures 2–4 for each of these analyses (except for the bile acids). Combined, the 4 GC-MS methods allowed us to identify 179 and quantify a total of 85 compounds. Table 4 shows the identified polar, organic acid extracts and bile acids (127 in total), Table 5 shows the identified volatile metabolites (52 in total) while Table 6 shows the 85 fully quantified compounds from all 4 techniques. These numbers actually represent the highest number of urine metabolites both identified and quantified by GC-MS to date. As seen in Table 1, previous GC-MS studies have reported up to 258 unique compounds being identified (but none quantified) [83] and approximately 95 compounds quantified in human urine [84]. Relative to NMR (see previous section) and other methods used to analyze human urine (Table 1), it appears that a multi-pronged GC-MS analysis is an excellent approach to characterize this biofluid. However, unlike NMR where nearly all detectable peaks are identifiable, metabolite coverage by GC-MS tends to be relatively incomplete. As seen in Figure 2, only 60% of the peaks could be identified using as reference the 2008 NIST library and other home-made GC-MS metabolite libraries. Likewise, in Figure 3, we see that only 65% of the organic acid peaks could be identified while in Figure 4, just 60% of the volatile compound peaks could be identified.

**Table 4:** List of metabolites identified in human urine extracts by GC-MS (except volatile compounds).

Amino acids	HMDB ID	Organic acids	HMDB ID
Beta-Alanine^a^	HMDB00056	Azelaic acid^b^	HMDB00784
Glycine^a^	HMDB00123	Benzoic acid^b^	HMDB01870
L-Alanine^a^	HMDB00161	Butyric acid^a^	HMDB00039
L-Asparagine^a^	HMDB00168	Cis-Aconitic acida,b	HMDB00072
L-Aspartic acid^a^	HMDB00191	Citraconic acid^b^	HMDB00634
L-Glutamine^a^	HMDB00641	Citramalic acid^b^	HMDB00426
L-Histidine^a^	HMDB00177	Citric acid^a^	HMDB00094
L-Isoleucine^a^	HMDB00172	Erythronic acid^a^	HMDB00613
L-Leucine^a^	HMDB00687	Ethylmalonic acid^b^	HMDB00622
L-Lysine^a^	HMDB00182	Gluconic acid^a^	HMDB00625
L-Ornithine^a^	HMDB00214	Glutaric acid^b^	HMDB00661
L-Phenylalanine^a^	HMDB00159	Glyceric acid^a^	HMDB00139
L-Serine^a^	HMDB00187	Glycolic acida,b	HMDB00115
L-Threonine^a^	HMDB00167	Glyoxalic acid^a^	HMDB00119
L-Tryptophan^a^	HMDB00929	Hippuric acida,b	HMDB00714
L-Tyrosine^a^	HMDB00158	Homovanillic acid^b^	HMDB00118
L-Valine^a^	HMDB00883	HPHPA^b^	HMDB02643
**Bile acids**	**HMDB ID**	Hydroxypropionic acid^b^	HMDB00700
Cholenic acid^c^	HMDB00308	Indoleacetic acid^b^	HMDB00197
Deoxycholic acid^c^	HMDB00626	Indolelactic acid b	HMDB00671
Lithocholic acid^c^	HMDB00761	Isobutyrylglycine^b^	HMDB00730
Ursodeoxycholic acid^c^	HMDB00946	Isocitric acid^a^	HMDB00193
**Misc**	**HMDB ID**	Isovalerylglycine^b^	HMDB00678
3-Methylhistidine^a^	HMDB00479	Kynurenic acid^b^	HMDB00715
5-Hydroxyindole^b^	HMDB59805	Levulinic acid^b^	HMDB00720
Creatinine^a^	HMDB00562	L-Lactic acid^b^	HMDB00190
Dimethylglycine^a^	HMDB00092	Malic acid^a^	HMDB00744
D-Xylitol^a^	HMDB02917	Malonic acida,b	HMDB00691
Glycerol 3-phosphate^a^	HMDB00126	m-Chlorobenzoic acid^b^	HMDB01544
Glycerol^a^	HMDB00131	m-Coumaric acid^b^	HMDB01713
Mannitol^a^	HMDB00765	Methylmalonic acid^b^	HMDB00202
Myoinositol^a^	HMDB00211	Methylsuccinic acid^b^	HMDB01844
Pseudouridine^a^	HMDB00767	o-Hydroxyhippuric acid^b^	HMDB00840
Scyllitol^a^	HMDB06088	o-Hydroxyphenylacetic acid^b^	HMDB00669
Sorbitol^a^	HMDB00247	Oleic acid^b^	HMDB00207
Urea^a^	HMDB00294	Oxalic acid^a^	HMDB02329
**Organic acids**	**HMDB ID**	Oxoglutaric acid^b^	HMDB00208
2,4-Dihydroxybutanoic acid^a^	HMDB00360	Palmitic acid^b^	HMDB00220
2-Ethylhydracrylic acid^b^	HMDB00396	Pantothenic acid^b^	HMDB00210
2-Furoylglycine^b^	HMDB00439	Phosphoric acid^b^	HMDB02142
2-Hydroxy-2-methylbutyric acid^b^	HMDB01987	p-Hydroxyphenylacetic acid^a^	HMDB00020
2-Hydroxyglutaric acid^a^	HMDB00694	Pimelic acid^b^	HMDB00857
2-Hydroxyvaleric acid^a^	HMDB01863	Pyroglutamic acida,b	HMDB00267
2-Ketobutyric acid^b^	HMDB00005	Pyruvic acid^b^	HMDB00243
2-Methyl-3-hydroxybutyric acid^b^	HMDB00354	Quinolinic acid^b^	HMDB00232
3,4-Dihydroxybenzeneacetic acid^b^	HMDB01336	Stearic acid^b^	HMDB00827
3,4-Dihydroxybutanoic acida,b	HMDB00337	Suberic acid^b^	HMDB00893
3,4-Dihydroxyhydrocinnamic acid^b^	HMDB00423	Succinic acida,b	HMDB00254
3-Aminoisobutanoic acida,b	HMDB03911	Sumiki’s acid^b^	HMDB02432
3-Hydroxyhippuric acid^b^	HMDB06116	Tartaric acid^a^	HMDB00956
3-Hydroxyisobutyric acid^a^	HMDB00023	Threonic acid^a^	HMDB00943
3-Hydroxyisovaleric acida,b	HMDB00754	Trans-Aconitic acid^a^	HMDB00958
3-Hydroxymethylglutaric acid^b^	HMDB00355	Trans-Ferulic acid^b^	HMDB00954
3-Hydroxyphenylacetic acid^b^	HMDB00440	Uric acid^a^	HMDB00289
3-Hydroxysebacic acid^b^	HMDB00350	Vanillyllactic acid^b^	HMDB00913
3-Methyladipic acid^b^	HMDB00555	Vanillylmandelic acid^b^	HMDB00291
3-Methylglutaconic acid^b^	HMDB00522	**Sugars**	**HMDB ID**
4-Deoxythreonic acida,b	HMDB02453	D-Fructose^a^	HMDB00660
4-Hydroxybenzoic acid^b^	HMDB00500	D-Galactose^a^	HMDB00143
4-Hydroxyphenyllactic acid^b^	HMDB00755	D-Glucose^a^	HMDB00122
5-Hydroxyhexanoic acid^b^	HMDB00525	D-Xylose^a^	HMDB00098
5-Hydroxyindoleacetic acid^b^	HMDB00763	D-Xylulose^a^	HMDB01644
Adipic acida,b	HMDB00448	L-Arabinose^a^	HMDB00646
Alpha-Hydroxyisobutyric acid^b^	HMDB00729	L-Fucose^a^	HMDB00174
Aminomalonic acid^a^	HMDB01147	Sucrose^a^	HMDB00258
Ascorbic acid^a^	HMDB00044		

a Polar metabolites.

b Targeted organic acids.

c Targeted bile acids. HPHPA: 3-(3-hydroxyphenyl)-3-hydroxypropanoic acid.

**Table 5:** Compound names, HMDB identification numbers, unique masses, mean mass spectral match quality, retention times, and NIST retention indices for volatile compounds in urine analyzed by HS-SPME-GC/MS.

Peak #	Compound	HMDB ID	Uniquemass	MSmatch	RT(min)	RI(Calc)	RI(NIST)
1	3-Methyl sulfolane	HMDB59667	68	867	0.83	725	654
2	3-Hexanone	HMDB00753	43	781	0.99	732	788
3	2-Pentanone	HMDB34235	43	894	1.61	758	654
4	1-Hydroxy-2-pentanone	HMDB59678	43	780	1.74	763	897
5	Allyl methylsulphide	HMDB31653	88	793	1.83	767	701
6	Dimethyl disulfide	HMDB05879	94	940	2.26	785	763
7	4-Heptanone	HMDB04814	71	919	3.34	831	883
8	1-Methylcyclohexanol	HMDB59693	71	821	3.81	850	931
9	2-Hexanone	HMDB05842	43	821	3.82	851	889
10	3,4-Dimethylthiophene	HMDB29304	111	906	4.15	865	888
11	Diallyl sulphide	HMDB36491	45	827	4.86	895	848
12	5-Methyl-2-hexanone	HMDB31549	43	816	5.29	913	836
13	1,3-Dithio cyclohexane	HMDB31473	120	841	5.85	936	1027
14	Dimethyl trisulfide	HMDB13780	126	873	6.01	943	950
15	Phenol	HMDB00228	94	859	6.34	957	998
16	o-Cymene	HMDB37050	119	928	7.19	993	1014
17	p-Cymene	HMDB05805	119	928	7.33	998	1018
18	m-Cymene	HMDB13806	119	928	7.43	1009	1022
19	1,4-Cineol	HMDB31455	111	912	8.71	1057	1016
20	p-Cresol	HMDB01858	107	930	8.99	1068	1082
21	Linalool oxide	HMDB31440	59	783	10.49	1131	1074
22	Iso-menthol	HMDB35764	57	923	10.99	1152	1141
23	Alpha-p-dimethylstyrene	HMDB29641	132	915	11	1153	1088
24	L-Menthol	HMDB03352	71	924	11.18	1160	1140
25	Undecane	HMDB31445	57	910	11.35	1168	1100
26	Ledene Oxide (II)	HMDB60295	43	743	11.54	1176	1201
27	Salicylic acid methyl ester	HMDB34172	120	857	11.65	1180	1193
28	Beta-Carvone	HMDB35824	82	859	12.81	1229	1247
29	Piperitone	HMDB34975	150	873	12.96	1235	1184
30	o-Thymol	HMDB35770	135	904	13.9	1275	1305
31	Beta-Cyclocitrol	HMDB41011	41	884	14.1	1283	1219
32	4,7-Dimethyl-benzofuran	HMDB59897	146	861	14.4	1296	1244
33	Cuminal	HMDB02214	133	861	14.82	1313	1235
34	2,6,10,10 Tetramethyl-oxa-spiro-4,5-dec-6-ene	HMDB36823	138	911	15.91	1359	1370
35	4-(2,6,6-Trimethyl-1-cyclohexen-1-yl)-2-butanone	HMDB32913	121	795	15.97	1362	1433
36	1,2,3,4-Tetrahydro-1,1,6-trimethyl naphthalene	HMDB59826	159	855	16.02	1364	1349
37	Alpha-Cedrene	HMDB59695	119	821	16.1	1367	1415
38	1,2,3,4,Tetrahydro-1,5,7-trimethylnapthalene	HMDB59696	159	795	16.59	1388	1310
39	1,2-Diydro-1,1,6-trimethyl-napthalene	HMDB40284	157	947	16.82	1398	1354
40	Beta-Guaiene	HMDB38157	161	879	17	1405	1500
41	Beta-Damascenone	HMDB13804	69	889	17.35	1420	1365
42	2,5-Cyclohexadiene-1,4-dione-2,6-di-tert-butyl	HMDB13817	177	843	18.51	1469	1462
43	Himachalene	HMDB38168	119	823	18.62	1473	1489
44	4-(2,6,6-Trimethylcyclohexa-1,3-dienyl)-but-3-en-2-one	HMDB37139	136	883	18.73	1478	1497
45	1-(2,6,6-Trimethyl-1-cyclohexen-1-yl)-1-penten-3-one	HMDB38130	121	788	19.05	1491	1507
46	2,4-Bis (1,1-Dimethylethyl)-phenol	HMDB13186	191	863	19.5	1510	1519
47	1-(2,3,6-Trimethyl phenyl)-3-Buten-2-one	HMDB59697	132	918	19.8	1523	1528
48	L-Calamenene	HMDB59910	159	920	20.25	1533	1534
49	Beta-Vatirenene	HMDB59676	202	806	20.45	1550	1545
50	1,6,7-Trimethylnaphthalene	HMDB59701	170	899	20.82	1564	1559
51	Azulol	HMDB59686	183	823	21.18	1581	1572
52	3,3,5,6-Tetramethyl-1-indanone	HMDB59683	173	686	21.28	1585	1579

**Table 6:** Concentrations of metabolites in human urine as measured by GC-MS.

Compounds	Observed Concentration(µM/mM creatinine)	Literature Values(µM/mM creatinine)
2,4-Dihydroxybutanoic acid	1.0 (0.3–1.8)	2.0±2.0
2-Ethylhydracrylic acid	2.1 (1.3–2.9)	NA**
2-Furoylglycine	3.6 (0.7–5.8)	9.95 (2.0–18.66)
2-Hydroxy-2-methylbutyric acid	0.8 (0.4–1.5)	2.8±1.5
2-Methyl-3-hydroxybutyric acid	4.0 (1.3–6.2)	5.5 (0.0–11.0)
3,4-Dihydroxybenzeneacetic acid	0.9 (0.6–1.3)	1.16±0.50
3,4-Dihydroxybutanoic acid	42.2 (21.9–56.1)	34.9 (12.5–57.2)
3-Aminoisobutanoic acid	25.8 (1.4–155.6)	2.91–116.43
3-Hydroxyhippuric acid	6.1 (4.1–7.8)	2.5 (0.2–5.0)
3-Hydroxyisobutyric acid	29.0 (11.8–59.8)	17.5 (2.0–33.0)
3-Hydroxyisovaleric acid	7.4 (4.1–17.2)	8.5±3.2
3-Hydroxymethylglutaric acid	3.2 (1.1–5.2)	3 (0–10)
3-Hydroxyphenylacetic acid	5.6 (1.4–15.0)	6.1±4.1
3-Hydroxysebacic acid	2.9 (1.7–3.8)	NA**
3-Methyladipic acid	2.1 (0.9–5.6)	2.9 (0.7–10.5)
3-Methylglutaconic acid	6.2 (2.8–8.3)	4.5 (0.0–9.0)
4-Deoxythreonic acid	18.2 (5.6–23.9)	25±10
4-Hydroxybenzoic acid	2.4 (1.3–3.6)	1.8±1.8
4-Hydroxyphenyllactic acid	2.8 (2.6–3.0)	1.1 (0.2–2.6)
5-Hydroxyhexanoic acid	2.7 (0.8–5.7)	3.5 (0.0–7.0)
5-Hydroxyindoleacetic acid	2.9 (0.4–5.8)	2.4±0.40
Adipic acid	3.0 (1.0–12.9)	5.1 (0.8–35.0)
Alpha-Hydroxyisobutyric acid	2.9 (1.3–4.6)	3.8
Azelaic acid	1.9 (1.2–3.2)	4.8 (1.3–15.0)
Benzoic acid	3.6 (1.4–7.5)	4.2 (1.9–6.5)
Cholenic acid	0.015 (0.004–0.030)	NA**
Cis/Trans-Aconitic acid	15.3 (5.2–21.8)	NA**
Citraconic acid	1.6 (0.9–2.1)	2.2
Creatinine*	12,246±8,369	10,090±310
Deoxycholic acid	0.055 (0.050–0.060)	NA**
D-Galactose	3.3 (1.3–8.5)	4.4 (0.0–31.0)
D-Glucose	35.6 (10.3–56.7)	31.12
D-Xylitol	2.3 (0.9–8.2)	3.50±2. 37
D-Xylulose	19.7 (6.4–32.6)	NA**
Ethylmalonic acid	2.4 (0.9–4.0)	2.0 (0.5–3.9)
Gluconic acid	13.3 (8.2–26.4)	NA**
Glutaric acid	1.4 (0.5–2.3 )	1.3 (0.6–2.6)
Glyceric acid	14.4 (7.8–28.8)	4.5 (0.0–9.0)
Glycine	75.7 (21–205)	151 (233–248)
Glycolic acid	39.4 (2.9–78.1)	35 (18–55)
Hippuric acid	217 (28–610)	257 (20–770)
Homovanillic acid	4.3 (0.9–8.9)	4.9±3.4
HPHPA	4.2 (0.9–15.3)	0.55–54.93
Hydroxypropionic acid	8.1 (3.1–11.8)	6.5 (3.0–10.0)
Indoleacetic acid	2.6 (0.6–5.4)	2.0±1.0
Indolelactic acid	0.9	0.460 (0.098–0.980)
Isocitric acid	56.8 (19.4–119.1)	58 (36–84)
Kynurenic acid	2.6 (0.9–4.1)	1.34±0.30
L-Arabinose	8.8 (0.8–19.4)	8.75 (6.12–11.84)
L-Asparagine	9.5 (3.0–26.0)	10.0 (4.6–16.3)
Levulinic acid	1.7 (0.6–2.5)	1.28 (0.33–2.75)
Lithocholic acid	0.16 (0.10–0.20)	NA**
L-Lactic acid	7.1 (0.9–16.4)	12.3±6.2
L-Ornithine	5.3 (2.0–8.8)	5.0 (2.0–8.0)
L-Phenylalanine	7.8 (5.0–11.3)	4.50±1.15
L-Serine	19.0 (10.4–35.8)	26.0±10.0
L-Tyrosine	15.1 (6.1–23.2)	8.8±4.5
L-Valine	5.5 (2.7–9.8)	3.0±1.0
Malonic acid	2.3 (1.2–3.1)	1 (0–2)
m-Chlorobenzoic acid	0.7	NA**
Methylmalonic acid	1.3 (0.5–2.1)	1.6 (0.0–3.6)
Methylsuccinic acid	4.7 (1.1–6.2)	2.3 (0.8–10.8)
Myoinositol	12.6 (5.1–15.3)	18.8±2.9
o-Hydroxyphenylacetic acid	2.9 (1.4–3.7)	7.71±3.65
Oxalic acid	8.2 (3.9–14.0)	1.11–33.34
Oxoglutaric acid	3.4 (1.4–12.5)	2.98 (0.42–18.30)
Palmitic acid	6.7 (2.6–24.3)	11 (6–23)
Phosphoric acid	784 (425–1170)	1000–4900
p-Hydroxyphenylacetic acid	6.0 (2.4–9.7)	9.68
Pimelic acid	1.9 (0.5–3.5)	NA**
Pseudouridine	29.8 (21.8–47.3)	26.02±4.62
Pyroglutamic acid	18.6 (4.5–24.9)	28.8 (3.4–54.2)
Pyruvic acid	2.1 (1.0–3.7)	2.13 (0.54–8.67)
Quinolinic acid	3.9 (0.9–15.1)	2.5±1.1
Scyllitol	3.6 (1.9–6.9)	NA
Sorbitol	3.9 (1.9–5.1)	3.50±2.24
Stearic acid	3.9 (2.3–7.7)	2.9 (1.6–6.6)
Suberic acid	1.2 (0.5–1.9)	0.5 (0.0–2.9)
Succinic acid	6.2 (2.5–13.5)	5.6±3.8
Sumiki’s acid	1.6	NA**
Threonic acid	14.8 (8.6–36.1)	10.0±7.0
Trans-Ferulic acid	1	36.3±11.2
Uric acid	186 (93–329)	188 (79–296)
Ursodeoxycholic acid	0.022 (0.013–0.030)	NA**
Vanillylmandelic acid	1.6 (0.6–2.8)	1.2±0.5

* Concentration of metabolite (Mean ± SD) is expressed by µM. HPHPA: 3-(3-hydroxyphenyl)-3-hydroxypropanoic acid; NA: not available; NA**: not available for healthy adults.

Incomplete compound identification is a common problem with global or untargeted GC-MS metabolomics. This may be due to any number of factors including spectral overlap due to incomplete separation, poor signal to noise for low intensity peaks, the lack of reference GC-MS spectral data for certain metabolites (especially unusual dietary sources), or the presence of spectral artefacts such as derivatization by-products or degraded metabolites in the GC-MS spectrum. For our GC-MS studies we used the NIST library supplemented with a home-made GC-MS reference library of known urine compounds assembled from the Human Metabolome Library [16]. No doubt the use of other commercially available reference GC-MS libraries such as the Fiehn GC-MS library from Agilent or the GOLM metabolome database library [85] might have allowed us to further increase our coverage. Likewise the use of a faster scan rate and/or a more sensitive GC-TOF instrument (instead of a slower scanning quadrupole GC-MS) certainly would have increased overall coverage.

Nearly all of the non-volatile metabolites (87) identified by our GC-MS analyses were also identified by NMR. Some of the exceptions were oxalic acid, phosphate and uric acid, each of which was detected by GC-MS but not by NMR. These compounds do not have NMR-detectable protons at physiological pH, making them essentially “NMR invisible”. Other compounds seen by GC-MS but not by NMR included metabolites that were generally below the detection limit of NMR (∼2 µM/mM creatinine) such as indolelactic acid and 2,4-dihydroxybutanoic acid. For our non-targeted GC-MS analysis, the lower limit of detection was 1 µM/mM creatinine (for 2,4-dihydroxybutanoic acid), while for our targeted organic acid GC-MS analysis the lower limit of detection was 0.7 µM/mM creatinine (for m-chlorobenzoic acid). Overall, our data suggests that the sensitivity of a standard single quadrupole GC-MS instrument is perhaps 1.5–2X better than a 500 MHz NMR instrument for water-soluble metabolites. It is also important to note that the level of water-soluble, non-volatile metabolite coverage obtained by GC-MS is not as great as seen with NMR (127 cmpds vs. 209 cmpds). The limited coverage of GC-MS is partly due to the fact that not all compounds can be readily extracted, easily derivatized or routinely separated on a GC column. Furthermore, when analyzing urine by GC-MS there is a need to pretreat the sample with urease (to reduce urea levels) that can diminish the abundance of some metabolites [86]. While GC-MS may not be the best method for analyzing water-soluble metabolites, it certainly excels at the detection of volatile metabolites. Indeed, only one of the volatile metabolites identified by GC-MS is identified by NMR (phenol). This certainly underlines a key strength of GC-MS relative to other metabolomics platforms. When comparing NMR to GC-MS we found that NMR is capable of detecting 121 compounds that the 4 combined GC-MS methods cannot detect while the combined GC-MS methods can detect 91 compounds that NMR cannot routinely detect. Overall, these data suggest that GC-MS and NMR appear to be complementary methods for the identification and quantification of small molecules in urine.

Among the 58 metabolites quantified by both GC-MS and NMR we found very good overall agreement, with the majority of measured concentration values falling within 20 ± 11% of each other. The concentration patterns and rankings of the most abundant to the least abundant compounds were also largely identical for the two platforms. A total of 12 metabolites exhibited somewhat larger concentration discrepancies between GC-MS and NMR (i.e; L-arabinose, L-serine (lower in GC-MS vs. NMR), 4-hydroxybenzoic acid and tyrosine (higher in GC-MS vs NMR). Some of these concentration differences may be due to the extraction or derivatization process needed to conduct GC-MS analyses. This can lead to unspecified compound losses, unusual derivatives or unrecognized fragmentation patterns. Therefore we would have expected at least a few GC-MS concentration values to be slightly lower than those seen by NMR. Likewise, it is important to remember that there are inherent errors (5–10%) in measuring peak areas (i.e. compound concentrations) both in GC-MS and NMR due to peak overlap, uneven baselines and spectral noise.

Nearly all of the compounds we detected or quantified in human urine by GC-MS have been previously described or mentioned in the GC-MS literature. One compound (scyllitol), however, appears not to have been previously detected by GC-MS. The identification of this compound by our GC-MS method was aided by its prior identification by NMR (see previous section). Additionally, a careful literature analysis also indicated the scyllitol is a normal constituent of human urine and has previously been detected in human urine via other methods.

As we noted with our NMR studies earlier, there are a few previously reported GC-MS detectable metabolites in human urine that appear to be artefacts. These artefactual metabolites may arise from extractions with different solvents, pre-treatment with urease, and chemical derivatization. For example, Shoemaker et al [84], reported the presence of bisethane in human urine. We also detected bisethane, but it appears to be artefact of chemical derivatization and is not a urine metabolite.

### Quantification and Identification of Urine Metabolites – Combined Direct Flow Injection and LC-MS/MS Assay

Direct flow injection (DFI) MS/MS or DFI-MS/MS is another commonly used global metabolic profiling method [87]. When isotopic standards are used along with multiple reactions monitoring (MRM), it is also possible to perform targeted metabolomics with very accurate concentration measurements. For our urine studies, we employed a combined DFI/LC-MS/MS approach, based on the commercially available AbsoluteIDQ p180 Kit (BIOCRATES Life Sciences AG, Innsbruck). When applied to urine, we were able to identify and quantify a total of 127 metabolites or metabolite species, including 34 acylcarnitines, 21 amino acids, 15 biogenic amines, creatinine, hexose, 35 phospatidylcholines, 15 sphingomyelins and 5 lysophosphatidylcholines. The amino acids and biogenic amines are analyzed by an LC-MS/MS method, whereas all other metabolites are analyzed by DFI-MS/MS as indicated in Table 7. DFI-MS/MS identifies lipid species (as opposed to specific lipids) using their total acyl/alkyl chain content (i.e. PC (38∶4)) rather than their unique structure. Therefore each lipid species identified by the BIOCRATES kit typically corresponds to 5–10 possible unique lipid structures. Consequently, the total number of phosphatidylcholines, sphingolipids and lysophosphatidylcholines structures identified by this method was 458, 19 and 6, respectively. Therefore, combining these probable lipid structures (483 in total, based on the known fatty acid and lipid composition in human serum) with the other 72 confirmed non-lipid metabolites, the DFI-MS/MS method yields 555 confirmed and probable metabolites or metabolite structures. All of these compounds, along with their corresponding estimated concentrations have all been entered into the UMDB.

**Table 7:** Concentrations of acylcarnitines, amino acids, biogenic amines, lysophosphatidylcholines, phosphatidylcholines and sphingomyelins in human urine by combined DFI/LC-MS/MS (BIOCRATES kit).

Acylcarnitines (DFI-MS/MS)	Mean (µM/mM creatinine)	Biogenic amines (LC-MS/MS)	Mean (µM/Mm creatinine)
Acetyl-L-carnitine	2.0 (0.4–5.9)	Kynurenine	0.41 (0.08–1.31)
Butenyl-L-carnitine	0.014 (0.008 –0.021)	L-Dopa	0.02 (0.01–0.04)
Butyryl-L-carnitine	0.76 (0.12–1.81)	Methioninesulfoxide	0.31 (0.14–0.66)
Decadienyl-L-carnitine	0.10 (0.04–0.30)	Phenylethylamine	0.011 (0.003–0.067)
Decanoyl-L-carnitine	0.04 (0.02–0.06)	Putrescine	0.20 (0.04–0.40)
Decenoyl-L-carnitine	0.14 (0.06–0.32)	Sarcosine	1.6 (0.2–9.4)
Dodecanoyl-L-carnitine	0.02 (0.01–0.04)	Serotonin	0.08 (0.04–0.12)
Dodecenoyl-L-carnitine	0.028 (0.011–0.042)	Symmetric dimethylarginine	2.8 (2.0–9.4)
Glutaconyl-L-carnitine	0.04 (0.01–0.06)	**Lysophosphatidylcholines (DFI-MS/MS)**	**Mean (µM/Mm creatinine)**
Glutaryl-L-carnitine	0.04 (0.03–0.10)	lysoPC a C16∶0	0.014
Hexadecadienyl-L-carnitine	0.0010 (0.0005–0.0040)	lysoPC a C17∶0	0.0032 (0.0008–0.0087)
Hexadecanoyl-L-carnitine	0.0022 (0.0020–0.0030)	lysoPC a C18∶0	0.0040 (0.0013–0.0100)
Hexanoyl-L-carnitine	0.05 (0.03–0.07)	lysoPC a C20∶4	0.0020 (0.0005–0.0038)
Hexenoyl-L-carnitine	0.019 (0.009–0.050)	lysoPC a C6∶0	0.0045 (0.0022–0.0074)
Hydroxyhexadecanoyl-L-carnitine	0.0015 (0.0006–0.0050)	**Phosphatidylcholines (DFI-LC/MS)**	**Mean (µM/Mm creatinine)**
Hydroxypropionyl-L-carnitine	0.008 (0.004–0.012)	PC aa C28∶1	0.006 (0.003–0.020)
Hydroxytetradecadienyl-L-carnitine	0.003 (0.001–0.008)	PC aa C30∶2	0.0005 (0.0001–0.0022)
Hydroxytetradecenoyl-L-carnitine	0.0020 (0.0008–0.0040)	PC aa C32∶0	0.0035 (0.0013–0.0071)
L-Carnitine	4.5 (0.6–15.2)	PC aa C32∶3	0.0009 (0.0003–0.0025)
Malonyl-L-carnitine	0.02 (0.01–0.04)	PC aa C34∶1	0.0077 (0.0023–0.0500)
Methylglutaryl-L-carnitine	0.030 (0.016–0.052)	PC aa C34∶2	0.0047 (0.0016–0.0240)
Methylmalonyl-L-carnitine	0.09 (0.05–0.16)	PC aa C34∶3	0.002
Nonayl-L-carnitine	0.20 (0.07–0.46)	PC aa C34∶4	0.0023 (0.0002–0.0180)
Octadecadienyl-L-carnitine	0.0007 (0.0003–0.0020)	PC aa C36∶1	0.0034 (0.0006–0.0250)
Octanoyl-L-carnitine	0.05 (0.03–0.09)	PC aa C36∶2	0.0160 (0.0021–0.0450)
Octenoyl-L-carnitine	0.50 (0.08–1.20)	PC aa C36∶3	0.0024 (0.0005–0.0240)
Pimelyl-L-carnitine	0.03 (0.01–0.11)	PC aa C36∶4	0.0018 (0.0005–0.0083)
Propenoyl-L-carnitine	0.003 (0.001–0.006)	PC aa C36∶6	0.0037
Propionyl-L-carnitine	0.07 (0.01–0.20)	PC aa C38∶3	0.0056
Tetradecadienyl-L-carnitine	0.0023 (0.0010–0.0050)	PC aa C38∶4	0.0022 (0.0005–0.0065)
Tetradecanoyl-L-carnitine	0.018 (0.001–0.088)	PC aa C38∶5	0.0007 (0.0002–0.0020)
Tetradecenoyl-L-carnitine	0.003 (0.001–0.006)	PC aa C38∶6	0.0010 (0.0005–0.0036)
Tiglyl-L-carnitine	0.10 (0.05–0.20)	PC aa C40∶3	0.00035 (0.0–0.00140)
Valeryl-L-carnitine	0.220 (0.037–0.440)	PC aa C42∶2	0.0009 (0.0003–0.0024)
**Creatinine**	**Mean**	PC aa C42∶4	0.0008 (0.0003–0.0025)
Creatinine*	12,475±7,955	PC ae C34∶1	0.0030 (0.0003–0.0110)
**Hexose**	**Mean (µM/Mm creatinine)**	PC ae C34∶2	0.0012 (0.0002–0.0075)
Hexose	110 (37–501)	PC ae C36∶1	0.0046 (0.0012–0.0080)
**Amino acids (LC-MS/MS)**	**Mean (µM/Mm creatinine)**	PC ae C36∶2	0.0016 (0.0002–0.0050)
Glycine	101.0 (37.0–250.6)	PC ae C36∶3	0.0009 (0.0001–0.0040)
L-Alanine	21.7 (7.2–47.4)	PC ae C36∶4	0.0022
L-Arginine	7.4 (3.2–14.6)	PC ae C38∶2	0.0015 (0.0007–0.0023)
L-Asparagine	8.8 (4.6–17.7)	PC ae C38∶3	0.0020 (0.0012–0.0028)
L-Aspartic acid	10.9 (1.9–26.8)	PC ae C38∶4	0.0006 (0.0–0.0024)
L-Citrulline	0.8 (0.2–1.7)	PC ae C38∶6	0.0065 (0.0027–0.0170)
L-Glutamic acid	6.4 (0.6–17.5)	PC ae C40∶2	0.0012 (0.0006–0.0020)
L-Glutamine	39.9 (18.4–72.5)	PC ae C40∶5	0.0006 (0.0001–0.0025)
L-Histidine	45.6 (21.7–71.5)	PC ae C42∶3	0.0007 (0.0001–0.0020)
L-Isoleucine	1.3 (0.5–2.7)	PC ae C44∶3	0.0010 (0.0002–0.0031)
L-Leucine	3.0 (1.6–6.0)	**Sphingomyelins (DFI-MS/MS)**	**Mean (µM/Mm creatinine)**
L-Lysine	17.9 (3.6–56.1)	SM (OH) C14∶1	0.0028 (0.0008–0.0049)
L-Methionine	0.8 (0.4–1.6)	SM (OH) C16∶1	0.0006 (0.0001–0.0023)
L-Ornithine	4.4 (1.2–22.1)	SM (OH) C22∶1	0.0023 (0.0009–0.0058)
L-Phenylalanine	5.9 (2.8–10.8)	SM (OH) C22∶2	0.0007 (0.0001–0.0028)
L-Proline	1.1 (0.1–2.5)	SM (OH) C24∶1	0.0010 (0.00034–0.0025)
L-Serine	24.5 (12.3–50.2)	SM C16∶0	0.0100 (0.0016–0.0700)
L-Threonine	14.6 (6.6–29.3)	SM C16∶1	0.0009 (0.0004–0.0023)
L-Tryptophan	5.6 (9.3–2.1)	SM C18∶0	0.0024 (0.0011–0.0070)
L-Tyrosine	8.7 (3.0–20.1)	SM C18∶1	0.0004 (0.0001–0.0013)
L-Valine	4.3 (2.4–10.4)	SM C20∶2	0.0003 (0.0–0.0016)
**Biogenic amines (LC-MS/MS)**	**Mean (µM/Mm creatinine)**	SM C22∶3	0.0011 (0.0008–0.0014)
Acetyl-ornithine	1.3 (0.5–2.8)	SM C24∶0	0.0085 (0.0047–0.0200)
Alpha-aminoadipic acid	7.2 (2.5–16.4)	SM C24∶1	0.0030 (0.0007–0.0120)
Asymmetric dimethylarginine	2.7 (1.4–4.2)	SM C26∶0	0.0014 (0.0002–0.0043)
Carnosine	1.2 (0.2–4.1)	SM C26∶1	0.0005 (0.0001–0.0012)
Dopamine	0.4 (0.2–0.7)		
Histamine	0.03 (0.01–0.10)		
Hydroxykynurenine	0.15 (0.06–0.34)		

* Concentration of metabolite (Mean±SD) is expressed by µM. aa: diacyl; ae: acyl-alkyl.

Our results show very good agreement with the previous studies conducted by BIOCRATES on human urine samples (Biocrates Application Note 1005-1). We found that the lower limit of quantification by DFI MS/MS based on the Absolute*IDQ* kit was 0.1 nM/mM creatinine for certain phosphatidylcholine species (i.e. PC aa C40∶3) and 0.1 nM/mM creatinine for certain sphingomyelin species (i.e. SM 26∶1). Comparison of our lipid results with literature data was difficult as relatively few papers report urine lipid concentration data. Indeed, the presence of lipids in urine is a little unexpected, but not entirely unreasonable. It is likely that urea, a well known chaotrope, facilitates the dissolution of small amounts of fatty acids and other lipid species in urine.

Many of the compounds we measured with this kit assay appear to be normal constituents of human urine but have not been previously reported (quantified and/or detected) in the scientific literature (with the exception of the BIOCRATES Application Note). In total, 53 compounds are being reported here for the first time as being normal constituents of human urine, while 68 compounds are being robustly quantified in human urine for the first time. The vast majority of these compounds are lipids.

Based on our results, the combined DFI/LC-MS/MS method detected 98 compounds or compound species that GC-MS and NMR methods could not detect, while GC-MS detected 161 compounds and NMR detected 181 compounds that DFI/LC-MS/MS could not detect. The 3 methods were able to detect a common set of 17 compounds including creatinine, L-glutamine, L-tryptophan, L-tyrosine and L-valine. Interestingly, the concentrations measured by DFI/LC-MS/MS, NMR and GC-MS (across the shared set of 17 compounds) showed generally good agreement (within 19 ± 7% of each other). The relatively small overlap, in terms of compound coverage, between the 3 platforms is a bit of a surprise and certainly serves to emphasize the tremendous chemical diversity that must exist in urine. Overall, by combining these 3 platforms, we were able to identify 295 and quantify 231 unique or non-overlapping metabolites or metabolite species. These data suggest that DFI/LC-MS/MS, GC-MS and NMR are highly complementary techniques for the identification and quantification of metabolites in human urine.

### Quantification and Identification of Urinary Trace Metals – ICP-MS

To determine the trace elemental composition of urine, we used inductively coupled plasma mass spectrometry (ICP-MS). ICP-MS is widely considered to be one of the best techniques for the characterization of the trace element composition of biological samples [88], [89]. Indeed, none of the other methods (^1^H-NMR, GC-MS and DFI/LC-MS/MS) are suited for measuring trace element composition or concentrations. Our multi-elemental analysis of urine using ICP-MS provided quantitative results for a total of 40 metals or trace minerals (Table 8). Based on their frequency of occurrence and overall abundance, all 40 trace elements appear to be normal constituents of human urine. Of these, 2 have previously not been quantified for healthy adults.

**Table 8:** Concentrations of ions (µmol/mmol creatinine) as determined by ICP-MS.

Compound Name	HMDB	Concentration (µM/mM creatinine)	Litterature values(µM/mM creatinine)
Aluminum (Al)	HMDB01247	0.31 (0.05–1.21)	0.667±0.037
Arsenic (As)	HMDB02290	0.10 (0.01–0.52)	0.16±0.16
Barium (Ba)	HMDB04142	0.0070 (0.0001–0.0655)	0.0129–0.0320
Cadmium (Cd)	HMDB03638	0.0004 (0.0002–0.0010)	0.00060 (0.00035–0.00100)
Calcium (Ca)	HMDB00464	200.0 (16.9–520.0)	299.0±99.0
Cerium (Ce)	HMDB13672	0.00048 (0.00008–0.00180)	0.0 – 0.0007
Cesium (Cs)	HMDB13669	0.0039 (0.0005–0.0110)	0.0061±0.0011
Chromium (Cr)	HMDB00599	0.0084 (0.0001–0.0460)	0.0100±0.0065
Cobalt (Co)	HMDB00608	0.0014 (0.0001–0.0140)	0.000810±0.00053
Copper (Cu)	HMDB00657	0.0163 (0.0006–0.1099)	0.0190 (0.0092–0.038)
Gallium (Ga)	HMDB01478	0.0056 (0.0012–0.0150)	0.0003–0.0006
Hafnium (Hf)	HMDB29201	0.00031 (0.00006–0.00110)	0.00027±0.00012
Iron (Fe)	HMDB00692	0.0890 (0.0025–0.4579)	0.11 (0.0–0.14)
Lanthanum (La)	HMDB13711	0.00020 (0.00003–0.00079)	0.00052±0.00039
Lead (Pb)	HMDB04628	0.0026 (0.0007–0.0110)	0.011 (0.0–0.020)
Lithium (Li)	HMDB05949	0.5 (0.2–0.9)	0.504 (0.058–3.414)
Magnesium (Mg)	HMDB00547	262 (42–1,189)	289.0±92.0
Manganese (Mn)	HMDB01333	0.013 (0.002–0.051)	0.011 (0.0–0.023)
Molybdenum (Mo)	HMDB01302	0.049 (0.006–0.295)	0.06±0.03
Neodymium (Nd)	HMDB13715	0.00017 (0.00004–0.00070)	0.0026±0.0013
Nickel (Ni)	HMDB02457	0.0080 (0.0015–0.0450)	0.00420 (0.00302–0.00572)
Palladium (Pd)	HMDB13670	0.0110 (0.0011–0.1410)	< 0.00001–0.2538
Platinum (Pt)	HMDB13671	0.000155 (0.000031–0.000560)	0.0000656
Posphorus (P)	HMDB01315	1,799 (221–4,936)	1,666.67–4,333.33
Potassium (K)	HMDB00586	3,593 (553–8,079)	4,605 (2,631–6,578)
Rhenium (Re)	HMDB13719	0.000096 (0.000013–0.000340)	NA
Rubidium (Rb)	HMDB01327	1.354 (0.187–3.673)	1.84±0.46
Ruthenium (Ru)	HMDB13668	0.0002 (0.0001–0.0009)	NA
Selenium (Se)	HMDB01349	0.149 (0.028–0.345)	0.11346 (0.07724–0.15010)
Sodium (Na)	HMDB00588	12,477 (1,863–37,249)	14,737 (5,263–36,842)
Strontium (Sr)	HMDB03642	0.163 (0.030–0.415)	0.1333
Tantalum (Ta)	HMDB04143	0.00020 (0.00004–0.00074)	0.00008±0.00006
Thallium (Tl)	HMDB13724	0.0003 (0.0001–0.0013)	< 0.00659
Thorium (Th)	HMDB29215	0.00020 (0.00002–0.00100)	< 0.00584
Tin (Sn)	HMDB01960	0.00110 (0.00016–0.00690)	< 0.00552
Titanium (Ti)	HMDB01966	0.0013 (0.0005–0.0030)	0.0142
Tungsten (W)	HMDB01989	0.0060 (0.0011–0.0280)	0.010±0.022
Vanadium (V)	HMDB02503	0.033 (0.004–0.083)	0.00162±0.00144
Zinc (Zn)	HMDB01303	0.460 (0.058–0.948)	0.52±0.25
Zirconium (Zr)	HMDB01475	0.021 (0.004–0.083)	0.0012–0.0074

As far as we are aware, this is the first multi-elemental study of urine that has been performed by ICP-MS. As seen in Table 8, there is generally good agreement between the values measured by ICP-MS and those previously reported in literature, with differences generally being less than 22 ± 10%. Larger differences are seen for gallium (Ga), lead (Pb), Neodymium (Nd), titanium (Ti) and vanadium (V), but these may be due to the effects of age, diet, local environment (minerals in local water) or diurnal variation. Alternately they may reflect real differences in the sensitivity or accuracy of the instruments being used. As a general rule, ICP-MS is considered as a gold standard for the identification and quantification of trace metals [18], so we would tend to place higher confidence in the values derived via ICP-MS over those measured by other technologies. By our measurements, the most abundant metals/salts are sodium (Na) (12.5 ± 10.6 mM/mM creatinine) and potassium (K) (3.6 ± 2.5 mM/mM creatinine) – as expected, while the least abundant was rhenium (Re) with a lower limit of quantification by ICP-MS of 96 pM/mM creatinine.

### Quantification and Identification of Urine Metabolites – Targeted HPLC Assays

The inventory of metabolites we detected and quantified by ^1^H NMR, GC-MS, DFI/LC-MS/MS and ICP-MS covers a significant portion of all chemical classes. However, these methods sometimes lack the necessary sensitivity, the appropriate instrumental configuration or detection capabilities and therefore fail to detect/quantify a variety of important compound classes. This includes a number of molecules that are normal constituents of urine such as thiols and isoflavones. To identify and quantify these 2 classes of metabolites we decided to employ High Performance Liquid Chromatography (HPLC). HPLC assays are the method of choice for detecting isoflavones and thiols as they are sensitive, precise and can be easily coupled with sensitive detection methodologies such as fluorescence and ultraviolet detection. In our studies, fluorescence and ultraviolet detection were used for the identification and quantification of urinary thiols and isoflavones, respectively.

Biological thiols, or mercaptans, are very active metabolic products of sulfur and play a central role in redox metabolism, cellular homeostasis and a variety of physiological and pathological processes. In urine, the most important thiols are L-cysteine and L-cysteinylglycine [90], [91], [92], [93]. Isoflavones or phytoestrogens form or constitute another important class of urinary metabolites [90]. Humans are exposed to these biologically active phytochemicals mainly through food intake via vegetables, fruit and wheat/bread products [94]. For the detection of thiols we developed assays to measure L-cysteine, L-cysteineglycine, L-glutathione and L-homocysyeine, while for isoflavones we developed assays to measure biochanin A, coumesterol, daidzein, equol, formonentin and genistein. Using these HPLC assays, we measured a total of 4 thiols and 6 isoflavones in urine (Table 9).

**Table 9:** Concentrations of isoflavones and thiols as determined by HPLC.

Compound Name	HMDB ID	Mean(µM/mM creatinine)	Oc (%)	Literature values (µM/mM creatinine)
Biochanin A^a^	HMDB02338	0.0088 (0.0027–0.0200)	77	NA
Coumesterol^a^	HMDB02326	0.0081 (0.0033–0.0120)	45	NA
Daidzein^a^	HMDB03312	0.026 (0.002–0.064)	88	0.036 ± 0.008
Equol^a^	HMDB02209	0.06 (0.01–0.10)	67	0.13 ± 0.12
Formonenetin^a^	HMDB05808	0.0007 (0.0002–0.0011)	33	NA
Genistein^a^	HMDB03217	0.0280 (0.0077–0.0640)	88	0.032 ± 0.007
L-Cysteine^b^	HMDB00574	81.0 (36.7–147.6)	100	33.4 ± 15.5
L-Cysteinylglycine^b^	HMDB00078	0.6 (0.28–1.54)	100	0.48 ± 0.15
L-Glutathione^b^	HMDB00125	0.030 (0.005–0.085)	100	0.065
L-Homocysteine^b^	HMDB00742	1.1 (0.6–1.7)	100	1.95 ± 1.47

a Isoflavone metabolites measured by HPLC/UV.

b Thiol metabolites measured by HPLC/FD.

Oc: occurrence;

NA: not available.

As seen in Table 9, there is generally good agreement between the values measured by these targeted HPLC assays and those previously reported in literature, with differences generally being less than ∼30%. Only one of these metabolites (L-cysteine) was measured independently on one of our other platforms (NMR) and the NMR concentration was found to be 25% lower than the HPLC assay. A possible explanation for this discrepancy is that cysteine measured by HPLC-FD yields total L-cysteine including the free form and L-cystine reduced to L-cysteine during the reaction [95]. On the other hand, NMR can distinguish between L-cysteine and L-cystine.

### The Composition of Human Urine – Comparison with Other Biofluids

By combining a systematic computer-aided literature survey with an extensive, quantitative multiplatform metabolomic analysis we have been able to comprehensively characterize the human urine metabolome. Our data suggests that there are *at least* 3079 detectable metabolites in human urine, of which 1350 have been quantified. At least 72 of these compounds are of microbial origin, 1453 are endogenous while 2282 are considered exogenous (note some compounds can be both exogenous and endogenous), coming from diet, drugs, cosmetics or environmental exposure. Using a chemical classification system developed for the HMDB [15] we found that human urinary metabolites fall into 230 different chemical classes (25 “super classes”, Table 2). Given that there are only 356 chemical classes in the entire human metabolome [16], this certainly demonstrates the enormous chemical diversity found in urine. As might be expected, most urinary metabolites are very hydrophilic, although there are clearly trace amounts of lipids and fatty acids that contribute a significant number of chemicals to the urinary metabolome (836 fatty acids and lipids). This is in rather striking contrast to the composition of serum [20] which is particularly rich in lipids (i.e. >17000 lipids and fatty acids). Relative to other biofluids such as CSF [18] or saliva [19], urine contains significantly more compounds (5–10X) and exhibits significantly more chemical diversity (2–3X). On the other hand, we know that every compound that is found in human urine is also found in human blood. In other words, the human urine metabolome is a subset of the human serum metabolome, both in terms of composition and chemical diversity [20]. However, more than 484 compounds we identified in urine (either experimentally or via literature review) were not previously reported to be in blood. The fact that so many compounds seem to be unique to urine likely has to do with the fact that the kidneys do an extraordinary job of concentrating certain metabolites from the blood. Consequently compounds that are far below the limit of detection in blood (using today’s instrumentation) are well above the detection limit in urine. In fact, concentration differences between the two biofluids sometimes exceed 1000X for certain compounds, such as histamine, androsterone, normetanephrine, testosterone 13, 14-dihydro-15-keto-PGE2, m-tyramine and aldosterone. So, while the number of water-soluble compounds in blood and urine may be almost identical, the concentrations of these compounds are often profoundly different. This difference, combined with the ability of the kidney to handle abnormally high or abnormally low concentrations of metabolites, makes urine a particularly useful biofluid for medical diagnostics. In fact, according to our data in the UMDB, urinary metabolites have been used to characterize nearly 220 diseases. Furthermore, the ability of the kidneys to filter toxins or xenobiotics makes urine a particularly useful biofluid for diet and drug monitoring and for assessing chemical or pollutant exposure [96].

### Method Comparison

One of the central motivations behind this work was to ascertain the strengths and weaknesses of several common metabolomic platforms for characterizing human urine. We employed 6 different analytical platforms: NMR; GC-MS; DFI/LC-MS/MS; HPLC/UV; HPLC/FD and ICP-MS. Using our literature-derived knowledge about the composition of human urine, along with custom-derived spectral libraries and targeted assays we were able to “push the limits” in terms of number of compounds that could be identified and/or quantified via each platform. In total, we identified 445 and quantified 378 distinct metabolites using these 6 different systems. According to Table 1, this is the largest number of urine metabolites ever identified and/or quantified in a single study. NMR spectroscopy was able to identify and quantify 209 compounds; GC-MS was able to identify 179 and quantify 85 compounds; DFI/LC-MS/MS identified and quantified 127 compounds; ICP-MS identified and quantified 40 compounds; while customized HPLC assays (with UV or FD detection) identified and quantified 10 compounds. The number of urinary metabolites we identified/quantified for NMR, GC-MS, DFI/LC-MS/MS and ICP-MS all represent “records” for these platforms.

**Figure 5:**
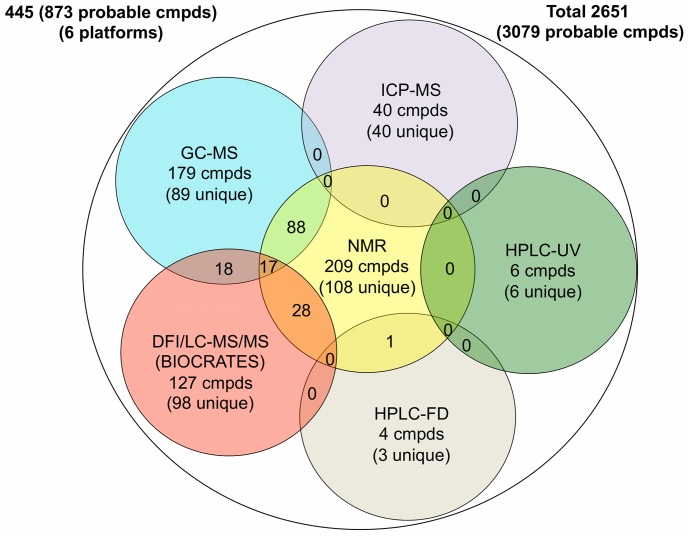
Venn diagram showing the overlap of urine metabolites detected by NMR, GC-MS, DFI/LC-MS/MS, ICP-MS, HPLC coupled to UV detection and HPLC coupled to fluorescence detection compared to the detectable urine metabolome.

In terms of platform overlap and compound complementarity, NMR and GC-MS were able to identify a common set of 88 metabolites; NMR and DFI/LC-MS/MS were able to identify and quantify a common set of 28 metabolites, while NMR, GC-MS and DFI/LC-MS/MS were able to identify a common set of 17 metabolites (15 amino acids, creatinine and hexose/glucose). All of these results are summarized in a Venn diagram (Figure 5). As might be expected, metabolite coverage differs from one analytical technique to another. These are difference mostly due to the intrinsic nature of the devices or platforms used. In particular, significant differences exist between these platforms in terms of their sensitivity or separation and/or extraction efficiency. Likewise the use of targeted vs. non-targeted methods along with issues related to compound stability, solubility and volatility led to some significantly different platform-dependent results.

Given that the known, quantifiable urine metabolome consists of ∼2651 known metabolites and metabolite species (corresponding to 3079 distinct structures), we can calculate that NMR is able to measure ∼8% (209/2651) of the human urine metabolome; GC-MS is able to measure ∼7% (179/2651); DFI/LC-MS/MS is able to measure ∼5% (127/2651); ICP-MS/MS is able to measure ∼1.5% (40/2651); HPLC/UV is able to measure ∼0.2% (6/2651); while HPLC/FD is able to measure ∼0.15% (4/2651) of the urine metabolome. When combined, the 6 analytical techniques are able to cover >16% of the known urinary metabolome (>445/2651). If we re-evaluate this fraction in terms of total metabolite structures (corresponding to known and highly probable metabolites), the urine metabolome consists of 3079 compounds. From this total we can calculate that NMR is able to measure ∼7% (209/3079) of the human urine metabolome; GC-MS is able to measure ∼6% (179/3079); DFI/LC-MS/MS is able to measure ∼18% (555/3079); ICP-MS/MS is able to measure ∼1.3% (40/3079); HPLC/UV is able to measure ∼0.2% (6/3079); while HPLC/FD is able to measure ∼0.13% (4/3079) of the urine metabolome. When combined, the 6 analytical techniques are able to cover >28% of the known and probable or putative urinary metabolome (>873/3079). In terms of chemical class coverage, NMR detects compounds from 15 of the 25 major chemical superclasses in urine, GC-MS detects compounds from 14 of the 25, DFI/LC-MS/MS detects 6 of the 25, ICP-MS detects 1 of the 25 while the targeted methods for thiols and isoflavones detect just 1 of each.

From these data we can conclude that NMR is currently the best method for identifying and quantifying urinary compounds. Not only does it permit measurement of the largest number of metabolites (209) but it also yields the greatest chemical diversity. Furthermore, NMR is non-destructive so that the same sample can be subsequently re-used for GC-MS, LC-MS or ICP-MS analyses. The minimal sample preparation and relatively rapid data collection for NMR also make it much more appealing for urine metabolomics, although the spectral analysis can be quite slow (∼1–2 hours per sample). While GC-MS is a close second in terms of overall coverage (179 metabolites, 14 chemical superclasses), these numbers represents the result of 4 different analyses performed on 2 different GC-MS instruments. Many labs would not have these multiple configurations available or the resources to routinely run these types of analyses. Likewise each sample required many hours of preparation, sample collection and data analysis. In this regard, multi-platform GC-MS is definitely not a high-throughput metabolomics technique. Relative to NMR and GC-MS, DFI/LC-MS/MS also performs well, with 127 compounds being quantified. However, DFI/LC-MS/MS provides very limited chemical diversity (only 6 chemical superclasses). On the other hand, DFI/LC-MS/MS requires very little sample volume (10 µL) and it is a very low-cost, largely automated, high-throughput route for measuring metabolites. The other techniques (HPLC, ICP-MS) we employed in this study, while useful, do not come close to matching the coverage or diversity of NMR, GC-MS or DFI/LC-MS/MS.

While we certainly went to considerable lengths to use current or cutting edge technologies to characterize the urine metabolome, it is also important to note that there is always potential for future improvement. Using higher field (900–950 MHz) NMR instruments, employing newer model GC-MS instruments or more sensitive GC-TOF instruments, using more than 3 derivatization or extraction steps for our GC-MS analyses, employing the latest version of the NIST database or a larger collection of GC-MS databases, implementing more sophisticated or targeted detection and separation techniques, using various commercial immunodetection kits or employing the latest LC-MS/MS techniques coupled to FT-MS or orbitraps – all of these could have added to the quantity and diversity of metabolites detected or quantified. However, like many laboratories, our resources are somewhat limited. Furthermore, in this study we wanted to address the question of how well a cross-section of commonly accessible metabolomic methods or platforms could perform in identifying and quantifying metabolites in urine.

While being able to quantitatively compare metabolite coverage and chemical diversity amongst the major metabolomics platforms (NMR, GC-MS and DFI/LC-MS/MS) is important, it is also useful to compare their consistency or reproducibility in terms of metabolite quantification. In particular we decided to assess the 3 major platforms in terms of their ability to identify and quantify a common group of compounds, namely the amino acids. Overall we found that the measured concentrations are in relatively good agreement (Table 10). However, a few exceptions are evident. For example, the NMR and DFI/LC-MS/MS concentrations of glycine and serine are higher than the GC-MS values (note that glycine exhibits the highest concentration among urinary amino acids). For serine, after the silylation reaction using MSTFA, we obtained serine-2TMS (13.9 min) and serine-3TMS derivatives (16.6 min). The chromatographic peak corresponding to serine-2TMS is weak and overlaps slightly with the urea peak. This overlap and the corresponding difficulty in peak integration may explain the quantitation differences compared to other analytical assays. Neither L-glutamine nor L-glutamic acid could be accurately quantified or identified by GC-MS. In our case, the glutamine peak co-elutes with glycerol-3-phosphate. Other investigators have noted that glutamine can also be hydrolyzed and converted to glutamic acid [97], or to pyroglutamic acid during derivatization [95]. As a result, only pyroglutamic acid could be identified in our GC-MS assay. The identification of glutamic acid and pyroglutamic acid can be complicated [97], which explains our failure to identify glutamic acid by GC-MS. L-arginine could not be detected by GC-MS, because it is converted to ornithine during derivatization [98], which probably explains the slightly higher concentration of ornithine measured by GC-MS, compared to the one determined by DFI/LC-MS/MS. Finally, L-cysteine and L-cystine can only be identified and quantified by NMR and targeted HPLC/FD because the identification and quantification of theses metabolites is not possible with the Biocrates kit. With GC-MS it has often been noted that L-cystine can be converted to L-cysteine during derivatization, while L-cysteine might be oxidized to L-cystine during prolonged storage of the standard solution [95], confounding the identification of L-cystine and L-cysteine.

**Table 10:** Comparison of urine concentrations of 23 quantified amino acids by different assays: DFI/LC-MS/MS (BIOCRATES kit), NMR and GC-MS.

Amino acids*	DFI/LC-MS/MS	NMR	GC-MS
Glycine	101.0±53.5	106±66	75.7±51.7
L-Alanine	21.7±10.6	21.8±10.3	NA
L-Arginine	7.4±3.0	8.6±3.6	NA
L-Asparagine	8.8±3.5	10.1±4.0	9.5±5.5
L-Aspartic acid	10.9±7.7	10.9±5.8	NA
L-Citrulline	0.8±0.4	NA	NA
L-Cysteine	NA	65.8±24.0	NA
L-Cystine	NA	12.5±5.4	NA
L-Glutamic acid	6.4±4.9	8.5±3.6	NA
L-Glutamine	39.9±14.3	37.2±14.8	NA
L-Histidine	45.6±15.6	43.9±19.5	NA
L-Isoleucine	1.3±0.4	1.3±0.5	NA
L-Leucine	3.0±1.0	2.8±0.9	NA
L-Lysine	17.9±12.2	17.2±11.5	NA
L-Methionine	0.8±0.3	1.4±0.6	NA
L-Ornithine	4.4±5.0	NA	5.3±1.9
L-Phenylalanine	5.9±2.0	6.4±2.3	7.8±2.0
L-Proline	1.1±0.6	NA	NA
L-Serine	24.5±9.2	25.3±9.7	19.0±7.0
L-Threonine	14.6±6.1	13.3±5.3	NA
L-Tryptophan	5.6±2.1	6.3±2.2	NA
L-Tyrosine	8.7±4.2	9.5±4.5	15.1±5.4
L-Valine	4.3±1.9	3.4±1.4	5.5±2.0

* Concentration (Mean ± SD) is expressed by µM/mM creatinine. NA: not available.

## Conclusion

Using a combination of multiple experimental assays supplemented with an extensive computer–assisted literature survey we were able to identify a total of 2651 metabolites or metabolite species (corresponding to 3079 distinct structures) that can be or have been identified and/or quantified in human urine using today’s technology. This information, which includes both normal and abnormal (disease or exposure-associated) metabolites has been placed into a publicly accessible web-enabled database called the Urine Metabolome Database (UMDB). To assess the validity of the literature data and to further investigate the capabilities of existing metabolomics technologies we conducted a comprehensive, quantitative analysis of human urine from 22 healthy volunteers. A total of 6 different platforms: NMR, GC-MS, DFI/LC-MS/MS, ICP-MS, HPLC/UV and HPLC/FD were used in this analysis. From this experimental work we were able to identify a total of 445 and quantify 378 metabolites or metabolite species. This corresponds to 873 unique structures (identified) and 806 unique structures (quantified). A total of 53 compounds or compound species are being reported here for the first time as being normal constituents of human urine, while 77 compounds or compound species are being robustly quantified in human urine for the first time. All of the metabolites that we experimentally identified and/or quantified have been added to the UMDB. Based on the information in the UMDB, our experimentally acquired data corresponds to 16% of the human urine metabolome (or 28% if we include probable or putative metabolites).

Considering the level of coverage, the diversity of chemical species and the ease with which analyses can be performed, we have determined that NMR spectroscopy appears to be the method of choice for global or untargeted metabolomic analysis of urine. On the other hand, the kit-based combined DFI/LC-MS/MS methods appear to be optimal for a targeted metabolomic approach. Using a multi-pronged GC-MS approach for urine metabolomics appears to be very promising in terms of coverage, but is not ideal for high-throughput analyses. All methods used in this study appear to be quite complementary with relatively little compound overlap. This strongly suggests that if sufficient time and resources are available, multiple methods should be used in urine metabolomic studies.

If additional resources had been available, we would have liked to assess other technologies (GCxGC-MS, FT-MS, isotope labeled-LC-MS) and to compare the level of metabolite coverage and chemical diversity attainable with these methods. However, this study is not intended to be the “final” word on urine or urine metabolome. Rather, it should be viewed as a starting point for future studies and future improvements in this field. Indeed, our primary objective for undertaking these studies and compiling this data was to help advance the fields of quantitative metabolomics, especially with regard to clinically important biofluids such as urine. Experimentally, our data should serve as a useful benchmark from which to compare other technologies and to assess coming methodological improvements in human urine characterization. From a clinical standpoint, we think the information contained in the human urine metabolome database (UMDB) should provide metabolomic researchers as well as clinicians and clinical chemists with a convenient, centralized resource from which to learn more about human urine and its unique chemical constituents.

## Methods

### Ethics Statement

All samples were collected in accordance with the ethical guidelines mandated by the University of Alberta as approved by the University’s Health Research Ethics Board. All individuals were over 18 years of age. All were approached using approved ethical guidelines and those who agreed to participate in this study were required to sign consent forms. All participants provided written consent.

### Collection of Urine Samples

Human urine samples (first pass, morning) were collected from 22 healthy adult volunteers (14 male, 8 female) in 120 mL sterile urine specimen cups. The average age of the volunteers was 30 (range 19–65) for females, and 32 (range 21–67) for males. Upon receipt (typically within 1 hour of collection), all samples were immediately treated with sodium azide to a final concentration of 2.5 mM. After centrifugation at 4000 rpm for 10 min to remove particulate matter, the urine samples were stored in 2 mL aliquots in falcon tube at −20°C until further use. Prior to each analysis, the samples were thawed at room temperature for 30 minutes and filtered for a second time via centrifugation.

### NMR Compound Identification and Quantification

All ^1^H-NMR spectra were collected on a 500 MHz Inova (Varian Inc., Palo Alto, CA) spectrometer using the first transient of the tnnoesy-presaturation pulse sequence. The resulting ^1^H-NMR spectra were processed and analyzed using the Chenomx NMR Suite Professional software package version 7.0 (Chenomx Inc., Edmonton, AB), as previously described [17]. Additional NMR spectra for 39 compounds were added to the Chenomx Spectral Reference Library using the company’s recommended spectral acquisition and formatting protocols. Further details on the NMR sample preparation, NMR data acquisition and the customized spectral library are provided in Method S1.

### GC-MS Compound Identification and Quantification

Twenty-two urine samples were extracted separately to obtain separate pools of polar, organic acid, bile acid (3 of the 22 urine samples were chosen for analysis and aliquots from these provided an additional “pooled normal” sample) and volatile metabolites using different protocols. The polar metabolites were extracted with cold HPLC grade methanol and double-distilled water after pretreatment with urease, followed by derivatization with MSTFA (N-Methyl-N-trifluoroacetamide) with 1% TMCS (trimethylchlorosilane). For organic acids, the ketoacids were converted first to methoxime derivatives, followed by derivatization with BSTFA (N,O-Bis(trimethylsilyl)trifluoroacetamide) after two successive extractions by ethyl acetate and diethyl ether. The bile acids were eluted with methanol through a SPE column (Bond Elute C18), followed by two different derivatization steps. First the bile acid extracts were esterified using 2% sulfuric acid in methanol, then after phase separation, the esterified bile acids were converted to the corresponding methyl ester-trimethylsilyl ether derivatives using MSTFA with 1% TMCS. Each set of extracted/derivatized metabolites (polar metabolites, organic acids, bile acids) was separated and analyzed using an Agilent 5890 Series II GC-MS operating in electron impact (EI) ionization mode.

The volatile compound extraction and analysis by GC-MS was far different from the other protocols. The pooled urine samples were acidified using HCl and transferred to a SPME (solid phase microextraction) vial (75 µm Carboxen/PDMS from Supelco), and then introducing the SPME fiber assembly into the GC-MS injector port according to procedures described elsewhere [99], [100]. The fibers were conditioned prior to use according to the manufacturer’s instructions by inserting them into the GC injector port. Further details on the extraction, derivatization, separation and GC-MS data analysis for the 4 separate groups of urine metabolites are provided in Method S2.

### Direct Flow Injection/LC-MS/MS Compound Identification and Quantification

To assess the performance of direct flow injection DFI-MS/MS methods in urine metabolomics and to determine the concentration ranges of a number of metabolites not measurable by other methods, we used the commercially available Absolute-IDQ p180 Kit (BIOCRATES Life Sciences AG - Austria). The kit, in combination with an ABI 4000 Q-Trap (Applied Biosystems/MDS Sciex) mass spectrometer, can be used for the targeted identification and quantification of 187 different metabolites or metabolite species including amino acids, biogenic amines, creatinine, acylcarnitines, glycerophospholipids, sphingolipids and hexoses. This method involves derivatization and extraction of analytes from the biofluid of interest, along with selective mass spectrometric detection and quantification via multiple reactions monitoring (MRM). Isotope-labeled internal standards are integrated into the kit plate filter to facilitate metabolite quantification. Metabolite concentrations were expressed as ratios relative to creatinine to correct for dilution, assuming a constant rate creatinine excretion for each urine sample (see Method S3 for additional information).

### Trace Element Analysis Using ICP-MS

Before trace elemental analysis by ICP-MS was performed, 22 urine samples were processed as described previously [101]. The concentrations of trace elements were determined on a Perkin-Elmer Sciex Elan 6000 quadrupole ICP-MS operating in a dual detector mode. Blank subtraction was applied after internal standard correction (see Method S4 for additional information). The accuracy of the ICP-MS analytical protocol was periodically evaluated via the analysis of certified reference standard materials (whole rock powders) BE-N and DR-N available from the SARM laboratory at the CRPG (Centre de Recherches Pétrographiques et Géologiques).

### Characterization of Isoflavones from Urine

We processed 22 urine samples as described previously [102], [103]. The isoflavones were isolated and concentrated by solid-phase extraction (Bond Elut C18 column). The elutes were hydrolyzed enzymatically as the urinary isoflavones occur predominantly as glucuronate and sulfate conjugates. The analysis were performed on an Agilent 1100 HPLC system using NovaPak C18 reversed-phase column connected to Agilent G1315B diode array detector with signals scanned between 190 and 400 nm (see Method S5 for additional information).

### Characterization of Thiols from Urine

To extract urinary thiols, we derivatized all 22 urine samples as described previously [104]. A mixture of reagent was used for the reduction and derivatization (with bromobimane) of thiols. The derivatized thiols were injected immediately into a hypersil-ODS HPLC Column connected to Agilent fluorometer operating at an excitation wavelength of 485 nm and emission wavelength of 510 nm (see Method S6 for additional information).

## Supporting Information

Method S1 NMR Compound Identification and Quantification.
(DOC)

Method S2 GC-MS Compound Identification and Quantification.
(DOC)

Method S3 Direct Flow Injection/LC-MS/MS Compound Identification and Quantification.
(DOC)

Method S4 Trace Element Analysis Using ICP-MS.
(DOC)

Method S5 Characterization of Isoflavones from Urine.
(DOC)

Method S6 Characterization of Thiols from Urine.
(DOC)
