# Supplementary material for: The Human Urine Metabolome
Source: PLoS One. 2013 Sep 4;8(9):e73076. doi: 10.1371/journal.pone.0073076 (PMC3762851; doi:10.1371/journal.pone.0073076)
Supplement: Method S1 — NMR Compound Identification and Quantification. (DOC) [file pone.0073076.s001.doc]

**Supporting Information, “The Human Urine Metabolome”**

Souhaila Bouatra, Farid Aziat, Rupasri Mandal, An Chi Guo, Michael R. Wilson, Craig Knox, Trent C. Bjorndahl, Ramanarayan Krishnamurthy, Fozia Saleem, Philip Liu, Zerihun T. Dame, Jenna Poelzer, Jessica Huynh, Faizath S. Yallou, Nick Psychogios, Edison Dong, Ralf Bogumil, Cornelia Roehring and David S. Wishart

## Method S1: NMR Compound Identification and Quantification

### NMR Sample Preparation: After thawing and centrifuging the crude urine, a 600 μL aliquot of urine was removed and placed in a 1.5 mL Eppendorf tube followed by the addition of 70 μL of D_2_O and 30 μL of a standard buffer solution (11.667 mM DSS [disodium-2,2-dimethyl-2-silapentane-5-sulphonate], 730 mM imidazole, and 0.47% NaN_3_ in H_2_O). The urine samples (700 μL) were then transferred to a standard 5 mm thin-walled glass NMR tube for subsequent NMR spectral analysis. In total, 22 urine samples were prepared in this manner. The pH of the samples ranged from 7.3–7.7.

### NMR Spectroscopy: All ^1^H-NMR spectra were collected on a 500 MHz Inova (Varian Inc., Palo Alto, CA) spectrometer equipped with a 5 mm HCN Z-gradient pulsed-field gradient (PFG) room-temperature probe. ^1^H-NMR spectra were acquired at 25 °C using the first transient of the Varian tnnoesy-presaturation pulse sequence, which was chosen for its high degree of quantitative accuracy . Spectra were collected with 128 transients and 8 steady-state scans using a 4 second acquisition time and a 1 second recycle delay.

NMR analysis: Prior to spectral analysis, all FIDs were zero-filled to 64,000 data points and line broadened 0.5 Hz. The methyl singlet produced by a known quantity of DSS was used as an internal standard for chemical shift referencing (set to 0 ppm) and for quantification. All ^1^H-NMR spectra were processed and analyzed using the Chenomx NMR Suite Professional software package version 7.0 (Chenomx Inc., Edmonton, AB), as previously described . Additional NMR spectra for the following compounds: 1,3-diaminopropane, 1-methyladenosine, 2-furoylglycine, 2-methylbutyrylglycine, 2-methylerythritol, 3-(3-hydroxyphenyl)-3-hydroxypropanoic acid, 3-hydroxyhippuric acid, 3-methylglutaconic acid, 4-deoxythreonic acid, 4-ethylphenol, 5-aminopentanoic acid, 7-methylxanthine, acetaminophen glucuronide, acetaldehyde, alpha-ketoisovaleric acid, asymmetric dimethylarginine, citraconic acid, citramalic acid, dehydroascorbic acid, desaminotyrosine, D-glucoronic acid, D-threitol, D-xylitol, D-xylose, erythritol, ethyl glucuronide, hydroxyproline, hydroxypropionic acid,, N-acetylneuraminic acid, p-cresol sulfate, phenylacetylglutamine, phenylglyoxylic acid, proline-betaine, pseudouridine, scyllitol, sorbitol, Sumiki's acid, symmetric dimethylarginine and syringic acid were added to the Chenomx Spectral Reference Library using the company’s recommended spectral acquisition and formatting protocols. This customized library was used to permit the identification and quantification of another 39 compounds to our urine spectra. Each spectrum was processed and analyzed by at least two experienced NMR spectroscopists to minimize compound mis-identification and mis-quantification. We also used sample spiking to confirm the identity of a number of ambiguously assigned compounds. Sample spiking involves the addition of 10-150 μM of the suspected compound to the sample of interest and inspection of the subsequent NMR spectra to determine if the expected NMR signals have changed. A total of 120 compounds were spiked, of which 70 compounds were confirmed to exist in human urine. Metabolite concentrations were expressed as ratios relative to creatinine to correct for dilution, assuming a constant rate creatinine excretion for each urine sample.

**References**

1. Saude EJ, Slupsky CM, Sykes BD (2006) Optimization of NMR analysis of biological fluids for quantitative accuracy. Metabolomics 2: 113-123.

2. Wishart DS, Lewis MJ, Morrissey JA, Flegel MD, Jeroncic K, et al. (2008) The human cerebrospinal fluid metabolome. J Chromatogr B 871: 164-173.
