# Supplementary material for: The Human Urine Metabolome
Source: PLoS One. 2013 Sep 4;8(9):e73076. doi: 10.1371/journal.pone.0073076 (PMC3762851; doi:10.1371/journal.pone.0073076)
Supplement: Method S2 — GC-MS Compound Identification and Quantification. (DOC) [file pone.0073076.s002.doc]

**Supporting Information, “The Human Urine Metabolome”**

Souhaila Bouatra, Farid Aziat, Rupasri Mandal, An Chi Guo, Michael R. Wilson, Craig Knox, Trent C. Bjorndahl, Ramanarayan Krishnamurthy, Fozia Saleem, Philip Liu, Zerihun T. Dame, Jenna Poelzer, Jessica Huynh, Faizath S. Yallou, Nick Psychogios, Edison Dong, Ralf Bogumil, Cornelia Roehring and David S. Wishart

**Method S2***:* **GC-MS Compound Identification and Quantification**

Extraction and derivatization of polar metabolites: Prior to analysis by GC-MS, 200 μL of 22 thawed urine samples containing 2 μL of ribitol in water (20 µg/mL) as an internal standard with 34 units of urease (Urease from Canavalia ensiformis (Jack bean), Sigma-Aldrich, Oakville, ON, Canada) was incubated at 37 °C for 30 min to deplete excess urea. Pretreatment was required to allow the detection of many other compounds . After the urease treatment an aliquot of 200 µL of urine were extracted with 800 µL of cold HPLC grade methanol and double-distilled water (8:1 vol/vol) and vortexed for 1 min. The sample was kept at 4 °C for 20 min and then centrifuged at 6,000 rpm for 8 min. After centrifugation, 200 µL of the supernatant were evaporated to dryness using a speedvac concentrator (Savant Instruments Inc., SDC-100-H, Farmingdale, NY). Extracted residues were reconstituted in 40 µL of 20 mg/mL methoxyamine hydrochloride (Sigma-Aldrich) in ACS grade pyridine and incubated at room temperature for 16 hours. Then 50 µL of MSTFA (N-Methyl-N-trifluoroacetamide) with 1% TMCS (trimethylchlorosilane) derivatization agent (Pierce) was added and incubated for 1 hour at 37 °C. The samples were vortexed twice during incubation to ensure complete dissolution. Derivatized samples were stored for at 4 ºC until analysis, which never exceed 48 hours.

Extraction and derivatization of organic acid metabolites: 1 mL of each 22 urine samples was incubated with 200 μL of methoxylamine hydrochloride solution at a concentration of 75 g/L at 60 °C for 30 min. to convert ketoacids to their methoxime derivatives (MO) . After the MO conversion, 3 drops of 6 N HCI were added to each sample and the pH was checked to insure that it was 1.0 or below. The mixture was extracted two times, the first time with 3 mL of ethyl acetate and the second time with 3 mL diethyl ether. The supernatant from the first and the second extraction was then combined. The resulting supernatant was decanted into a vial and put in a 37 °C heating block under nitrogen for complete evaporation. After evaporation, the residue was dissolved in 40 μL hexane and 30 μL of N,O-Bis(trimethylsilyl)trifluoroacetamide (BSTFA) was added. The resulting mixture was placed in a heating block at 60-90 °C for 10 min after which the BSTFA-supplemented extract was placed into a GC/MS autosampler vial using a transfer pipet.

Extraction and derivatization of bile acids: To extract the bile acids, 5 mL of 4 lyophilized urine samples are dissolved in 1 mL of double-distilled water. The urine samples passed through a Solid Phase Extraction (SPE) cartridge (Bond Elut C18 cartridges, 500 mg/6mL columns) which was obtained from J.T. Baker (Philipsburg, NJ, USA). Each cartridge was preconditioned with 2 mL of chloroform: methanol (2: 1) followed by 2 mL of methanol. The bile acids were recovered from the cartridge by elution with 6 mL of methanol. The bile acids were then derivatized first by evaporating the methanol extract and 1 mL of 2% sulfuric acid in methanol was added. The mixture was heated 1 hour in oven at 82-87 ºC. Then 500 µL of KCl was added and the mixture extracted two times with 2 mL hexane. After phase separation, the organic layer was evaporated to dryness. The esterified bile acids were converted into trimethylsilyl ethers using 50 µL of MSTFA (N-Methyl-N-trifluoroacetamide) with 1% TMCS.

GC-MS analysis: Derivatized extracts of polar, organic acid and bile acid metabolites were analyzed using an Agilent 5890 Series II GC-MS operating in electron impact (EI) ionization mode. A 2 µL aliquot was injected with a splitless ratio onto a 30 m × 0.25 mm × 0.25 µm DB-5 column (Agilent Technologies). For optimal separation, different column temperature programs were selected for generic polar, organic acid and bile acid metabolites. For the derivatized polar metabolites, the injector port temperature was held at 250 °C and the helium carrier gas flow rate was set to 1 mL/min at an initial oven temperature of 50 °C. The oven temperature was increased at 10 °C/min to 310 °C for a final run time of 45 min. For the derivatized organic acids, the injector port temperature was held at 250 °C and the helium carrier gas flow rate was set to 1 mL/min at an initial oven temperature of 70 °C. The oven temperature was increased at 6 °C/min to 310 °C for a final run time of 54 min. Finally, for the derivatized bile acid, the injector port temperature was held at 100°C for 5 min, ramped to 290 °C at 35 °C/min and held for 15 min for a final run time of 45 min. The full scan spectra (50-500 m/z; 1.7 scans/sec) were acquired after a 5 min solvent delay, with an MS ion source temperature of 200 °C.

Identification and quantification of metabolites was performed as previously described . Briefly, the Automated Mass Spectral Deconvolution and Identification System (AMDIS) spectral deconvolution software (Version 2.62) from NIST (National Institute of Standards and Technology) was used to process the total ion chromatogram and the EI-MS spectra of each GC peak. After deconvolution, the purified mass spectrum of each of the trimethylsilylated metabolites was identified using the NIST MS Search program (version 2.0d) linked to the 2008 NIST mass spectral library (2008). Retention Indices (RIs) were calculated using a C8-C20 and a C22-C40 alkane mixture solution (Fluka, Sigma-Aldrich) which served as an external alkane standard. Metabolites were identified by matching the EI-MS spectra with those of reference compounds from the NIST library. In AMDIS, each search produces a list of library spectra (“hits”), which is ranked by the similarity to the target spectrum according to a mathematically computed “match factor”. The match factor indicates the likelihood that our spectrum and the reference NIST spectrum arose from the same compound. In the current case, we considered hits with a match factor of >60% and a probability >20%. In addition, authenticity checks were performed by using additional published retention index libraries [51]. Peaks having no match to published retention indices and/or no match to the AMDIS/NIST GC/MS spectral library were identified by matching the experimental RI of each metabolite with an in-house RI library containing 312 TMS-derivatized human metabolites . Forty-five pure standards obtained from the Human Metabolome Library were run through the GC–MS (using the same protocol described above) to confirm their identity. Retention indices and EI spectra were subsequently used for producing external seven-point calibration curves (for absolute quantification). Metabolite concentrations were expressed as ratios relatives to creatinine to correct for dilution, assuming a constant rate creatinine excretion for each urine sample.

Analysis of volatile compounds: Frozen urine samples from 22 individuals were completely thawed at room temperature and 3 mL of urine from each of these 22 samples were pooled. Briefly, 5 mL aliquots of the pooled urine samples adjusted to pH 1–2 with 400 µL of HCl were transferred to 20 mL SPME head-space vial. After the addition of 0.8 g of NaCl and stirring (0.5 mm × 0.1 mm bar) at 800 rpm, the SPME vial was crimp-sealed with a PTFE septum and an aluminum cap (VWR Scientific, Canada). The addition of salt increased the extraction efficiency for many metabolites, especially the polar ones while increasing the sensitivity of an analytical methodology . The vial was placed on a hotplate-stirrer plate (VWR Scientific, Canada) set at 60 °C and 1000 rpm, and the SPME fiber assembly (75 µm Carboxen/polydimethylsiloxane [PDMS], Sigma-Aldrich, USA) was introduced according to procedures described elsewhere , and maintained above the solvent during the extraction. The fibers were conditioned prior to use according to the manufacturer's instructions by inserting them into the GC injector port. After a measured extraction time (60 min, as optimized), the SPME fiber was drawn into the needle, removed from the vial and inserted in the injector port (250 °C) of the GC–MS system for 3 min, where the analytes were thermally desorbed and transferred directly to the analytical column. A blank run corresponding to the analysis of the coating fiber not submitted to any extraction procedure was also carried out to check for any possible contamination of the fiber during sample preparation and analysis.

To fully analyze the urinary volatiles, a high-throughput SPME–GC–MS method was developed on an Agilent Technologies 6890A gas chromatograph–5973N mass spectrometer (GC–MSD, Agilent Technologies, Palo Alto, CA) equipped with a split/splitless injector operated in splitless mode using a Merlin Microseal system (septum and nut fitting Agilent system) and a narrow-diameter liner (Sigma-Aldrich, USA) specially designed for SPME sample introduction. The GC–MSD system was controlled by Agilent Technologies ChemStation (G1701CA) for data acquisition. Chromatography was performed on an HP-5 (30 m x 0.25 mm coated with 0.25 µm 5% diphenyl/95% dimethylpolysiloxane) column with helium as carrier gas (37 kPa), at a constant flow rate of 1.3 mL/min. The injector was held at 250 °C with a purge-off time of 1 min, a 50 mL/min split vent flow at 1.5 min and a gas saver flow of 20 mL/min at 3 min. The GC temperature program began at 50 °C, was held for 2 min, then raised to 150 °C at a rate of 7 °C/min, held for 2 min and finally ramped to 310 °C at a rate of 7 °C/min with a 2.84 min hold at the final temperature. The total run time was 47 min. The transfer line and MS source temperatures were maintained at 200 °C and 240 °C, respectively. Mass spectra in 30–400 m/z range were recorded using an electron impact source with a single quadrupole at a scanning speed of 2.8 scans/s, ionization energy of 70 eV with the electron multiplier set to auto tune procedure. Metabolite identification was matched through manual interpretation of spectra and matching against the Agilent MS ChemStation Software, equipped with NIST08 mass spectral library and were compared to the published retention indices for 5% diphenyl of 95% dimethylpolysiloxane GC column. All the compounds were manually assessed with respect to the mass spectral match and the assigned unique mass , , .

**References**

1. Shoemaker JD, Elliott WH (1991) Automated screening of urine samples for carbohydrates, organic and amino-acids after treatment with urease. J Chromatogr-Biomed 562: 125-138.

2. Tanaka K, Hine DG, Westdull A, Lynn TB (1980) Gas-chromatographic method of analysis for urinary organic-acids. I. retention indexes of 155 metabolically important compounds. Clin Chem 26: 1839-1846.

3. Wishart DS, Lewis MJ, Morrissey JA, Flegel MD, Jeroncic K, et al. (2008) The human cerebrospinal fluid metabolome. J Chromatogr B 871: 164-173.

4. Wishart DS, Knox C, Guo AC, Eisner R, Young N, et al. (2009) HMDB: a knowledgebase for the human metabolome. Nucleic Acids Res 37: D603-D610.

5. Camara JS, Marques JC, Perestrelo RM, Rodrigues F, Oliveira L, et al. (2007) Comparative study of the whisky aroma profile based on headspace solid phase microextraction using different fibre coatings. J Chromatogr A 1150: 198-207.

6. Arthur CL, Pawliszyn J (1990) Solid-phase microextraction with thermal-desorption using fused-silica optical fibers. Anal Chem 62: 2145-2148.

7. Jia CR, Luo YZ, Pawliszyn J (1998) Solid phase microextraction combined with HPLC for determination of metal ions using crown ether as selective extracting reagent. J Microcolumn Sep 10: 167-173.

8. Adams RP (2009) Identiﬁcation of essential oil components by gas chromatography/mass spectorscopy. Carol Stream, IL. 804 p.

9. Stein SE, Linstrom PJ, Mallard W (2009) NIST chemistry webBook, NIST standard reference database number 69. Gaithersburg: National Institute of Standards and Technology.

10. Robinson AL, Boss PK, Heymann H, Solomon PS, Trengove RD (2011) Development of a sensitive non-targeted method for characterizing the wine volatile profile using headspace solid-phase microextraction comprehensive two-dimensional gas chromatography time-of-flight mass spectrometry. J Chromatogr A 1218: 504-517.
