# Supplementary material for: The Human Urine Metabolome
Source: PLoS One. 2013 Sep 4;8(9):e73076. doi: 10.1371/journal.pone.0073076 (PMC3762851; doi:10.1371/journal.pone.0073076)
Supplement: Method S3 — Direct Flow Injection/LC-MS/MS Compound Identification and Quantification. (DOC) [file pone.0073076.s003.doc]

**Supporting Information, “The Human Urine Metabolome”**

Souhaila Bouatra, Farid Aziat, Rupasri Mandal, An Chi Guo, Michael R. Wilson, Craig Knox, Trent C. Bjorndahl, Ramanarayan Krishnamurthy, Fozia Saleem, Philip Liu, Zerihun T. Dame, Jenna Poelzer, Jessica Huynh, Faizath S. Yallou, Nick Psychogios, Edison Dong, Ralf Bogumil, Cornelia Roehring and David S. Wishart

**Method S3***:* **Direct Flow Injection/LC-MS/MS Compound Identification and Quantification**

A total of 22 urine samples were analyzed with the AbsoluteIDQ kit using the protocol described in the AbsoluteIDQ user manual. Briefly, urine samples were left to thaw on ice and then vortexed and centrifuged at 13,000 rpm. 10 µL of supernatant from each urine sample was loaded onto the kit’s filter paper substrate and dried under nitrogen. Subsequently, 20 uL of a 5% phenyl-isothiocyanate solution was added for derivatization and after 20 min incubation the filter spots were dried using a nitrogen evaporator. Extraction of the metabolites was then achieved using methanol containing 5 mM ammonium acetate. The extracts were analyzed using an ABI 4000 Q-Trap (Applied Biosystems/MDS Sciex) mass spectrometer equipped with a solvent delivery system. A standard flow injection protocol consisting of two 20 μL injections (one for the positive and one for the negative ion detection mode) was applied for all measurements. MRM detection was used for quantification. For the amino acids and biogenic amines a LC-MS/MS method in positive mode was applied as described in the kit user manual. The MetIDQ software was used to control the entire assay workflow, from sample registration to automated calculation of metabolite concentrations to the export of data into other data analysis programs. The kit was originally validated for plasma/serum samples but cross validation with other techniques (NMR, GC-MS) also showed excellent concordance with the kit’s results with urine samples.
