# Supplementary material for: The Human Urine Metabolome
Source: PLoS One. 2013 Sep 4;8(9):e73076. doi: 10.1371/journal.pone.0073076 (PMC3762851; doi:10.1371/journal.pone.0073076)
Supplement: Method S4 — Trace Element Analysis Using ICP-MS. (DOC) [file pone.0073076.s004.doc]

**Supporting Information, “The Human Urine Metabolome”**

Souhaila Bouatra, Farid Aziat, Rupasri Mandal, An Chi Guo, Michael R. Wilson, Craig Knox, Trent C. Bjorndahl, Ramanarayan Krishnamurthy, Fozia Saleem, Philip Liu, Zerihun T. Dame, Jenna Poelzer, Jessica Huynh, Faizath S. Yallou, Nick Psychogios, Edison Dong, Ralf Bogumil, Cornelia Roehring and David S. Wishart

**Method S4***:* **Trace Element Analysis Using ICP-MS**

In particular, a total of 2 mL (±0.1 mL) of urine sample was aliquoted and sonicated in an ultrasound water bath for 10 min in order to obtain a homogeneous dispersion. The sample was then diluted to 10 mL with 2% HNO_3_. Trace element concentrations were determined on a Perkin-Elmer Sciex Elan 6000 quadrupole ICP-MS operating in a dual detector mode. Blank subtraction was applied after internal standard correction. A four point calibration curve was used to quantify compounds (0, 0.025, 0.050, and 0.100 ppm for Na; 0, 0.25, 0.50, and 1.00 ppm for Ca, Mg, Fe, K; 0, 0.005, 0.010, and 0.020 ppm for the remaining elements). A typical count rate for a 10 ppb Pb solution would be 150,000 to 200,000 cps. The sample uptake rate was approximately 1 mL/min with 35 sweeps per reading using one reading per replicate and three replicates. Dwell times were 10 to 20 ms for all elements with the exception of as (which was 100 ms). The relative standard deviations (2σ level) for As, Ni, Pb, and Zn were between 5 and 10%.
