# Supplementary material for: The Human Urine Metabolome
Source: PLoS One. 2013 Sep 4;8(9):e73076. doi: 10.1371/journal.pone.0073076 (PMC3762851; doi:10.1371/journal.pone.0073076)
Supplement: Method S5 — Characterization of Isoflavones from Urine. (DOC) [file pone.0073076.s005.doc]

**Supporting Information, “The Human Urine Metabolome”**

Souhaila Bouatra, Farid Aziat, Rupasri Mandal, An Chi Guo, Michael R. Wilson, Craig Knox, Trent C. Bjorndahl, Ramanarayan Krishnamurthy, Fozia Saleem, Philip Liu, Zerihun T. Dame, Jenna Poelzer, Jessica Huynh, Faizath S. Yallou, Nick Psychogios, Edison Dong, Ralf Bogumil, Cornelia Roehring and David S. Wishart

**Method S6***:* **Characterization of Thiols from Urine**

20 μL of urine was treated with 10 μL of 4 mM NaBH4 (dissolved in a solution of 333 mL/L dimethyl sulfoxide and 66 mM NaOH), followed by 5 μL of a solution containing 2 mM EDTA and 2 mM DTT, then 5 μL of 1-octanol, and finally 5 μL of 2 mol/L HCl. The mixture was incubated for 1 min at 25 °C (to allow reduction of disulfides). 25 μL of N-ethylmorpholine buffer (2 mol/L, pH 8.0) and 10 μL of 25 mM bromobimane (in 1:1 acetonitrile/H2O, v/v) were added to the mixture. After incubation of the mixture for 1 min, 20 μL of the derivatized sample was injected immediately into HPLC Column connected to an Agilent fluorometer operating at an excitation wavelength of 365 nm and emission wavelength of 475 nm. The 150 × 4.6 mm Hypersil-ODS column was equilibrated with 30 mM ammonium nitrate and 40 mM ammonium formate buffer, pH 3.6 (buffer A). The thiols were eluted from the column with a linear gradient of acetonitrile (buffer B) (0-11% in 18 min at a flow rate of 1 mL/min). The column equilibration time was 5 min, and the column was run at ambient temperature. The retention time for each analyte was calculated using external calibrators at three different concentrations.
